# Complementary approaches to tooth wear analysis in Tritylodontidae (Synapsida, Mammaliamorpha) reveal a generalist diet

**DOI:** 10.1371/journal.pone.0220188

**Published:** 2019-07-25

**Authors:** Daniela C. Kalthoff, Ellen Schulz-Kornas, Ian Corfe, Thomas Martin, Stephen McLoughlin, Julia A. Schultz

**Affiliations:** 1 Department of Zoology, Swedish Museum of Natural History, Stockholm, Sweden; 2 Department of Human Evolution, Max Planck Institute for Evolutionary Anthropology, Leipzig, Germany; 3 Center of Natural History (CeNak), University of Hamburg, Hamburg, Germany; 4 Developmental Biology Program, Institute of Biotechnology, University of Helsinki, Helsinki, Finland; 5 Institut für Geowissenschaften und Meteorologie, Universität Bonn, Bonn, Germany; 6 Department of Palaeobiology, Swedish Museum of Natural History, Stockholm, Sweden; Ecole normale superieure de Lyon, FRANCE

## Abstract

Stereoscopic microwear and 3D surface texture analyses on the cheek teeth of ten Upper Triassic to Lower Cretaceous tritylodontid (Mammaliamorpha) taxa of small/medium to large body size suggest that all were generalist feeders and none was a dietary specialist adapted to herbivory. There was no correspondence between body size and food choice. Stereomicroscopic microwear analysis revealed predominantly fine wear features with numerous small pits and less abundant fine scratches as principal components. Almost all analyzed facets bear some coarser microwear features, such as coarse scratches, large pits, puncture pits and gouges pointing to episodic feeding on harder food items or exogenous effects (contamination of food with soil grit and/or dust), or both. 3D surface texture analysis indicates predominantly fine features with large void volume, low peak densities, and various stages of roundness of the peaks. We interpret these features to indicate consumption of food items with low to moderate intrinsic abrasiveness and can exclude regular rooting, digging or caching behavior. Possible food items include plant vegetative parts, plant reproductive structures (seeds and seed-bearing organs), and invertebrates (i.e., insects). Although the tritylodontid tooth morphology and auto-occlusion suggest plants as the primary food resource, our results imply a wider dietary range including animal matter.

## Introduction

Tritylodontids (Synapsida Osborn, 1903 [1], Mammaliamorpha Rowe, 1988 [2], Tritylodontidae Cope, 1884 [3]) are a common, widespread, abundant, and yet understudied component of Mesozoic terrestrial ecosystems. Their earliest records are from the latest Rhaetian (Late Triassic, around 202 Ma) including *Oligokyphus* sp. from Canada [4], or the less certainly dated *Oligokyphus triserialis* of Germany [5], which, although most likely of Rhaetian age (Late Triassic, 209–201 Ma), could be from any time within the Norian, Rhaetian or Hettangian (Late Triassic to Early Jurassic, ~227–199 Myrs) [6]. The last known fossils are from the Lower Cretaceous (Barremian to Aptian; 130–113 Ma) of Japan (*Montirictus* Matsuoka, Kusuhashi, and Corfe, 2016 [7]) and Siberia (*Xenocretosuchus* Tatarinov and Matchenko, 1999 [8]). This considerable timespan (some 69–114 Ma) and a Cretaceous last appearance indicate that tritylodontids were most likely the last surviving members of the non-mammaliaform synapsids, a group that corresponds approximately to the paraphyletic ‘mammal-like reptiles’ of earlier usage. With nearly 20 genera and 30 or more species described and generally considered valid, and known from hundreds if not thousands of individuals, they are certainly the most commonly found and well-known late surviving members of this group. To date, tritylodontids are known from Europe, Africa, Asia, North America, and Antarctica [9] but have never been reported from Australia, and likely have yet to be discovered in South America ([10] contra [11]). Body size in Tritylodontidae ranges from small, vole- to rat-sized taxa (the smallest are *Lufengia delicata* and then *Yunnanodon brevirostris*) to large, wolf- or capybara-sized taxa (*Kayentatherium wellesi* and *Bienotherium magnum*) [10, 12]. Given the considerable range of body sizes in Tritylodontidae, we expect the food choice of at least the small- and large-bodied taxa to be different with respect to dietary composition and biomechanical properties of the ingested food items.

When first described, tritylodontids were considered to be some of the earliest known mammals. As such, they were, with little discussion, included within non-ruminant Artiodactyla (the Middle Jurassic *Stereognathus*, [13]), as possible members of Marsupialia (based on the Early Jurassic *Tritylodon*, [14]), or Rodentia (again based on *Tritylodon*, [15]). Cope [3] proposed tritylodontids to be members of Multituberculata within Marsupialia (also based on *Tritylodon*), and Simpson [16], reviewing the evidence from additional tritylodontid taxa in considerable detail, agreed with the multituberculate position for tritylodontids but disagreed that Multituberculata should be placed within Marsupialia. Subsequent studies of multiple, more complete, specimens (e.g. first Young on *Bienotherium* [17], then Kühne on *Oligokyphus* [18]) provoked a large shift in opinion, mainly owing to the discovery of postdentary bones still attached to the mandibles, towards tritylodonts most likely being ‘mammal-like reptiles’ close to the origin of mammals [17, 18]. By phylogenetic definition, tritylodontids are basally divergent members of Mammaliamorpha [2], and their alternative phylogenetic placements result in altered compositions and membership of Mammaliamorpha. Although all cladistic analyses agree with Young and Kühne [17, 18] and place Tritylodontidae outside of Mammalia, their exact phylogenetic position is somewhat uncertain [19]. Some analyses suggest they are members of a primarily herbivorous non-mammalian synapsid radiation, Cynognathia [20–23]. However, most recent authors place them in the clade Probainognathia that includes Mammaliaformes and Mammalia, but not in Cynognathia. In these analyses, tritylodontids are recovered as close relatives, or the closest relatives, of Mammaliaformes, though their exact position is variable. As such, they have been considered either the sister clade to Mammaliaformes [2, 24, 25] (though the latter called the Mammaliaformes clade Mammalia), or located 2–5 nodes basally of Mammaliaformes in close proximity to *Sinoconodon*, *Adelobasileus*, Brasilodontidae, and Trithelodontidae variably arranged in close proximity [26–33].

The structure of the tritylodontid dentition, with auto-occluding multi-cuspid postcanine teeth with 6–11 principal cusps in the upper teeth and 4–6 in the lowers [13, 17, 18, 34], and procumbent and/or enlarged, potentially evergrowing incisors [14, 18, 35, 36], combined with the robust skull morphology and aspects of the reconstructed skull musculature [37], has led to much speculation about the diet of tritylodontids (S1 Fig). This began with the first publication on tritylodontids, by Owen [13] in which *Stereognathus ooliticus* was described. Concluding from the multi-cuspid and multi-rooted teeth that *Stereognathus* probably belonged to the non-ruminant artiodactyls, Owen stated that “the food of which, if we may judge from the existing hogs and peccaries, was of a mixed nature” ([13] p.4). Selby et al. ([38] p. 103), in summarizing the find and Owen’s description, modified this in stating that Owen considered that *Stereognathus* was “of omnivorous habits”.

However, omnivory may not have been Owen’s intended dietary hypothesis. Owen [13], after describing *Stereognathus*, stated that much of the significance of the specimen lay in the possibility of analysing the process by which the restoration of an unknown animal is made from a single fragment of jaw and teeth. In discussing this in depth with reference to the *Stereognathus* specimen, he did indeed hypothesise that “The broad sex-cuspid crowns of the molar teeth of the *Stereognathus* might crush vegetable matter or insect-cases” ([13] p.5). However, he proceeded to show that the teeth were unlike those of known insectivores and closer to those of extinct artiodactyls, and so “Just in the ratio of this resemblance, therefore, is the inclination to view the *Stereognathus* […] as having been herbivorous rather than insectivorous, and as having been most probably a mixed feeder” ([13] p.6). In this context, and despite the earlier suggestion of a similar diet to the omnivorous hogs and peccaries, it appears that ‘mixed feeder’ refers to a mixture of various kinds of plants and plant parts, rather than omnivory, i.e., eating food of both plant and animal origin. Whereas Cope [3] (p.691) stated that “the Tritylodontidae […] were also of herbivorous or granivorous habits”, and Hu et al. [39] (p.385) stated that their “new tritylodontid may have been omnivorous rather than herbivorous” when describing *Yuanotherium*, almost all other hypotheses of the diet of tritylodontids have, similarly to Owen [13], suggested herbivory [16, 18, 26, 37, 40, 41]. Despite these multiple dietary hypotheses, comparisons of the skulls and dentitions of tritylodontids to multituberculates and muroid rodents [35, 37], and the general consensus of herbivory, the diet of tritylodontids has never been formally analysed.

### Motivation of the study

Our motivation here, in the first study of tooth wear patterns of the Tritylodontidae is, to use stereomicroscopic dental microwear and 3D surface texture analyses in a sample of ten species of small-, medium-, and large-bodied tritylodontid taxa from the Late Triassic to Early Cretaceous, in order to (1) test their hypothesized adaptation to herbivory, (2) evaluate the influence of different body sizes on diet, and (3) identify possible candidates for fodder plants.

#### Hypotheses

The following two hypotheses guided our analysis:

1. All analyzed tritylodontid taxa subsisted on a predominantly herbivorous diet, i.e., primary and secondary food resources consist of living plants and/or shed plant parts.
2. Body size had an influence on food choice.

## Materials and methods

### Material

**Fig 1:**
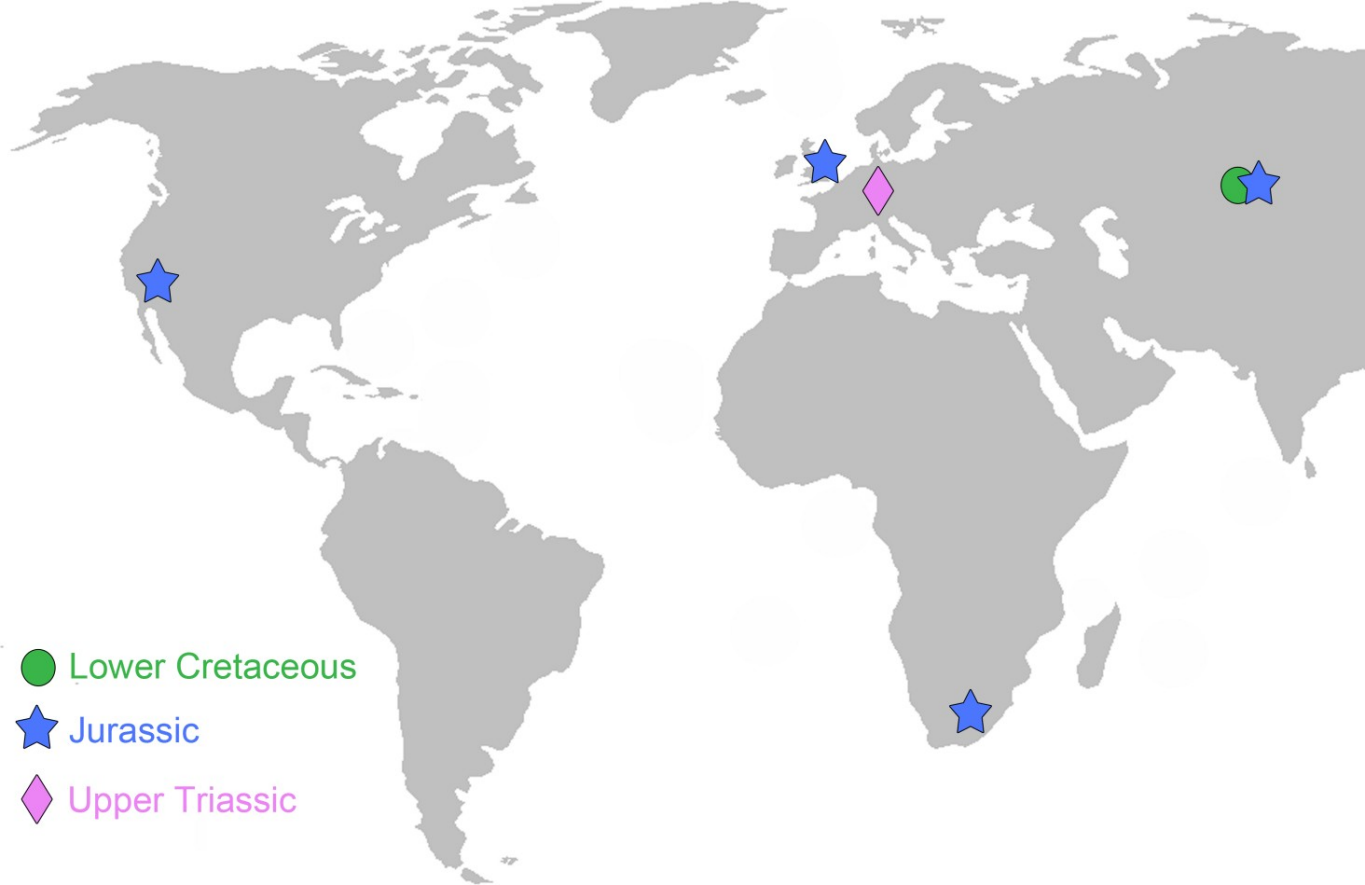
Approximate geographic locations and stratigraphic positions of the tritylodontid taxa analyzed in this paper. Map reprinted from Wikimedia Commons under a CC BY license, with permission from ‘E Pluribus Anthony’, original copyright 2006. The map is similar but not identical to the original map and is for illustrative purposes only.

We analyzed 39 upper (PC) and lower (pc) postcanine teeth of the following ten tritylodontid taxa: *Oligokyphus major*, *Oligokyphus* sp. (GB), *Oligokyphus* sp. (US), *Oligokyphus triserialis*, *Tritylodon longaevus*, *Stereognathus ooliticus*, *Stereognathus* cf. *ooliticus*, *Stereognathus* sp., *Stereognathus sibiricus*, *Dinnebitodon amarali*, cf. *Dinnebitodon amarali*, *Kayentatherium wellesi*. Stratigraphically, the samples span the Upper Triassic to Lower Cretaceous (S1 Table), and geographically, across the Northern Hemisphere and extending to South Africa (Fig 1, Table 1). Country codes refer to ISO 3166–1 alpha-2.

**Table 1:** Summary table of the studied taxa.

Taxon (specimens studied)	Body size estimation	Stratigraphic age	Locality
*Oligokyphus major* (2)	Small	Early Jurassic (Pliensbachian)	Windsor Hill, GB
*Oligokyphus* sp. (1)	Small	Early Jurassic (Hettangian)	Pant 4 quarry, St. Brides Island, GB
*Oligokyphus* sp. (1)	Small	Early Jurassic (Pliensbachian)	Kayenta Fm., US
*Oligokyphus triserialis* (1)	Small	Upper Triassic (Rhaetian)	Olgahain, DE
*Tritylodon longaevus* (1)	Large	Early Jurassic	South Africa
*Stereognathus ooliticus* (5)	Medium	Middle Jurassic (Early Bathonian)	Hornsleasow, GB
*Stereognathus* cf. *ooliticus* (1)	Medium	Middle Jurassic (Late Bathonian)	Old Cement Works Quarry, Kirtlington, Oxfordshire, UK
*Kayentatherium wellesi* (3)	Large	Early Jurassic (Pliensbachian)	Kayenta Fm., US
*Dinnebitodon amarali* (3)	Medium	Early Jurassic (Pliensbachian)	Kayenta Fm., US
*Stereognathus* sp. (14)	Medium	Middle Jurassic (Bathonian)	Itat Fm. (I2), Berezovsk Quarry, Krasnoyarsk, RU
*Stereognathus sibiricus* (7)	Medium	Early Cretaceous (Barremian-Aptian)	Ilek Fm., W1-Shestakovo 1, RU

Country codes refer to ISO 3166–1 alpha-2.

#### Body size estimations

Where possible, we chose specimens that were at least two-thirds of the size of the largest known individual, based on the width of the upper or lower teeth, to ensure that adults of each species were being compared. We also determined the three size categories used based on maximum known skull size for the species, and for species where skull size was not available, on maximum upper or lower tooth width. We follow Gaetano et al. [10] but with modifications for some species to take into account skull incompleteness and larger known individuals. Skull lengths of 0–99 mm, 100–199 mm and 200 mm+ were used for our categories of small-, medium-, and large-bodied tritylodontids. Tritylodontids analysed in the small category are *Oligokyphus* sp. (GB), *Oligokyphus* sp. (US), *Oligokyphus triserialis* and *Oligokyphus major*; in the medium category, *Stereognathus ooliticus*, *Stereognathus* cf. *ooliticus*, *Stereognathus* sp., *Stereognathus sibiricus*, *Dinnebitodon amarali*, cf. *Dinnebitodon amarali;* and in the large category, *Tritylodon longaevus* and *Kayentatherium wellesi* (S1 Table).

Although larger species, such as *Kayentatherium*, are considered to have had reptilian, non-determinant growth [37], recent work suggests smaller taxa, such as *Oligokyphus major*, may have followed a growth pattern previously unknown and in between that of most mammals and reptiles [42]. Nonetheless, we feel our delimitation of two-thirds adult size together with the three size categories ensure the majority of specimens used are probably adults, and adequately capture the range of size variation within tritylodontids. It should be noted that *Kayentatherium* and *Dinnebitodon*, in the large-bodied and medium-bodied size categories, respectively, had only juveniles with successful tooth microwear readings, so caution should be taken with interpretations of body size and diet in these taxa (see also discussion).

#### Acronyms to museum collections

Acronyms to museum collections: BRSUG: Geology Museum, University of Bristol, Bristol, GB; FMNH: Finnish Museum of Natural History, Helsinki, Finland; GLRCM: Gloucester City Museum, Gloucester, GB; GPIT: Paläontologische Sammlung, Fachbereich Geowissenschaften, Universität Tübingen, DE; MCZ: Museum of Comparative Zoology, Harvard, Massachusetts, USA; NHMUK: Natural History Museum, London, GB; USNM PAL: National Museum of Natural History, Department of Paleobiology, Washington D.C., USA; ZIN PH: Paleoherpetological collection of the Zoological Institute, Russian Academy of Sciences, Saint Petersburg, RU.

### Methods

A recent study [43] comparing tooth microwear patterns using stereomicroscopy and scanning electron microscopy (SEM) showed that the combination of the two approaches leads to a more robust and objective interpretation of feeding adaptations, especially in extinct taxa. Here, we applied a different combination of methods to analyze microwear (stereomicroscopy and 3D surface texture) for a sample of ten tritylodontid taxa spanning the Upper Triassic to Lower Cretaceous. Similar target areas on identical facets were analyzed by only one user per method (DCK for stereomicroscopy and ESK for 3D surface texture analysis). Both approaches have proven useful for recognizing species-specific differences in microscopic scars but they reveal evidence at very different magnifications, illumination, field of view (area analyzed), and parameters evaluated. Therefore, we did not attempt to directly compare the results of the two methods but interpreted them independently, then combined these interpretations to develop hypotheses concerning the feeding adaptations in tritylodontid taxa.

Wear features on all available genuine facets were analyzed regardless of tooth position. This approach was necessary owing to the frequent traces of postmortem alterations [44] and/or the rapid loss of the thin enamel layer [20] on the steep slopes of tritylodont postcanines. On this basis, 39 facets of 10 taxa could be used for this study.

Stereoscopic analysis of microwear was undertaken using a Zeiss Discovery V12 stereomicroscope set to a magnification of ×100 with a target area of 100 μm^2^, following the protocol defined by Solounias and Semprebon [45]. We used the following ten parameters:

- number of small pits (SP)
- number of large pits (LP)
- presence of large pits: 0 = absent or one LP, 1 = present (two or more LP)
- total number of pits (TP)
- number of fine scratches (FS)
- number of coarse scratches (CS)
- presence of coarse scratches: 0 = absent or one CS, 1 = present (two or more CS)
- total number of scratches (TS)
- presence of gouges (G): 0 = absent or one G, 1 = present (two or more G)
- presence of puncture pits (PP): 0 = absent or one PP, 1 = present (two or more PP).

**Fig 2:**
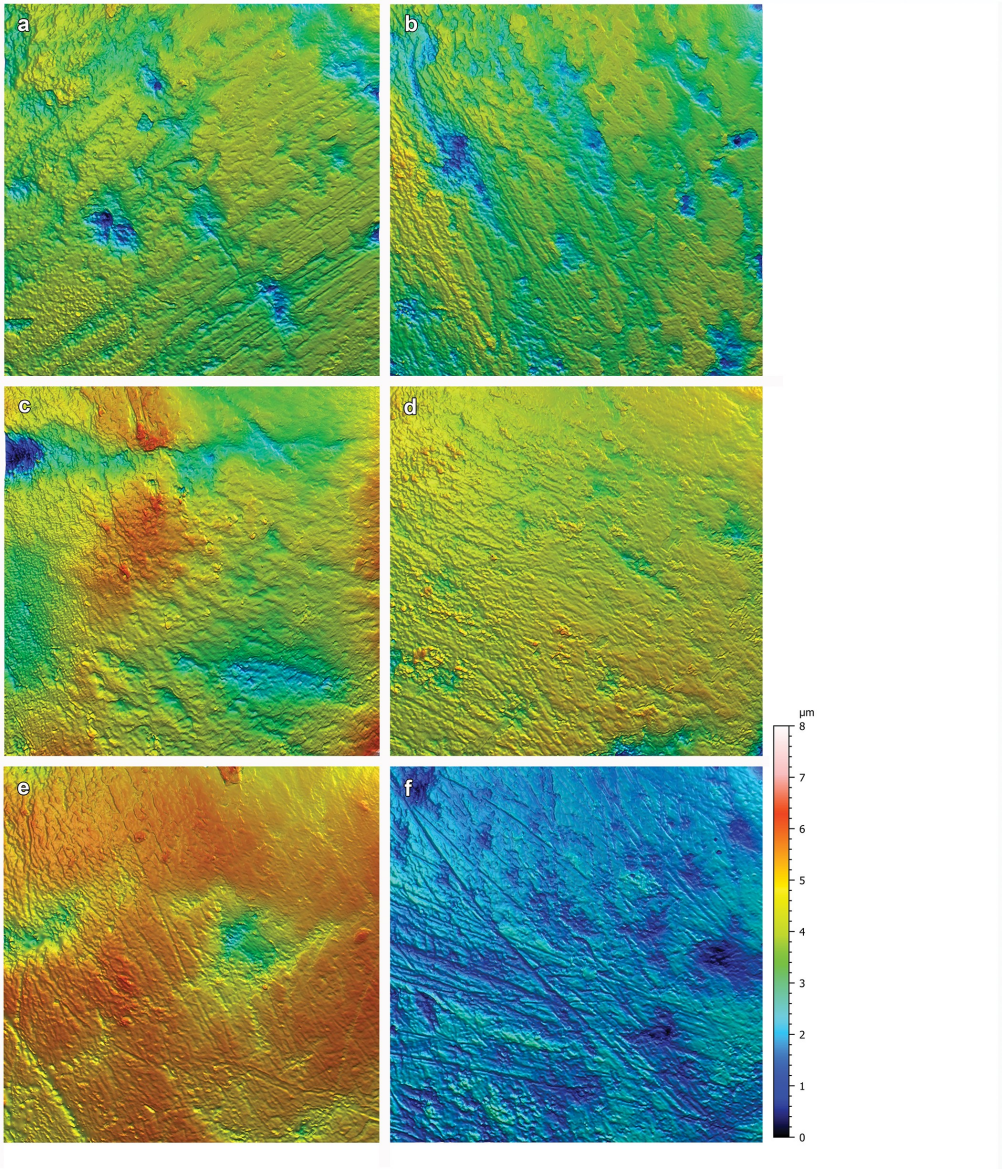
Surface texture (coloured) meshed axiomatic models for species used in the ANOVA. 3D top view normalized in XYZ, 100% resolution, tile size = 160x160 μm; (a) *Stereognathus ooliticus* (GLRCM 2104); (b) *Oligokyphus major* (BRSUG 26300); (c) *Stereognathus* sp. (ZIN PH 64/117); (d) *Stereognathus sibiricus* (ZIN PH 9/154); (e) *Kayentatherium wellesi* (MCZ 8811); (f) *Dinnebitodon amarali* (MCZ 8830–1). Color scale corresponds to height on the z-axis with red representing higher elevations and blue lower elevations.

3D surface texture analysis (according to ISO 25178; after [46]) with 30 parameters was executed with a disc-scanning surface instrument μsurf custom (Nanofocus, Oberhausen, Germany) set to a ×100 long distance objective with a target area of 160×160 μm (Fig 2). Scans with 95% or more of the surface measured were accepted. Each of the surface scans needed metrological pre-processing to reduce the nominal form (F-Operator, for more details see ISO 25178) and measurement noise (low pass S-Filter: median filter with filter size 5x5 and Gaussian filter with filter size 3x3; default cut offs are used) before parameters could be applied. We applied the following procedures using Mountains Map Premium v. 7.4.8076 analysis software (Digital Surf, Besançon, France): leveling (least square method), mirroring the y- and z-axes in the case of casts, and outlier removal (removal of isolated outliers and those around edges, with normal strength, removal of noise), fill in of non-measured points (smoothing method), and removal of form using a 2nd degree polynomial. From the meshed axiomatic 3D models, we chose the following four of the 30 ISO 25178 parameters for further analysis: closed dale area (*Sda*), pit void volume with p = 80% (*Vvv*), arithmetic mean peak curvature (*Spc*), and peak density (*Spd*) with Wolfpruning of 5%. 3D surface texture parameters are used to obtain an overall understanding of the damage to the surfaces, and were chosen to be representative for the feature and functional parameters of the ISO 25178 and to be potentially diagnostic of differences in surface features. We chose a set of four feature parameters as robust representatives for the feature microtopography responsible for trapping food particles indicating the area of the dales (*Sda*); the curvature of the peaks (*Spc*), indicating the density of the peaks (*Spd*), and one functional parameter quantifying the volume of the dales (*Vvv*). Initially, we checked all 30 ISO 25178 parameters. However, because many of the possible parameters are correlated, we chose four parameters (the same number as for stereoscopic microwear) that were complimentary to the absence/presence or count data in stereoscopic microwear analysis.

Statistical tests were performed using PAST v. 3.14 [47]. Differences between species were tested using one-way analysis of variances (ANOVA), which is a standard procedure for microwear data. Because surface texture data is hardly ever normally distributed and homoscedastic, we decided to adopt a comparative approach to stereoscopic microwear and rank-transform (*Spc*, *Spd*) or log-transform (*Sda*, *Vvv*) surface texture parameters. Tukey’s test was used to assess the numerical parameters for differences between species (only for species with n≥2). A Kruskal-Wallis test for equal medians was used for the non-numerical (presence/absence) parameters to identify significant differences between species. Principle component analysis (PCA) applying a variance-covariance matrix was used to visualize relatedness between taxa. We set the cut-off for the loadings to +/− 0.7; this allowed us to isolate the dominant use-wear features for each taxon.

## Results

### Stereomicroscopic microwear

The stereoscopic microwear results (Tables 2 and 3, S2 Table) revealed that wear features were predominantly fine with numerous small pits and less common fine scratches. One-way ANOVA results (species with N≥2) show significant differences in the number of small pits (SP) between eleven of 14 species pairs (Table 3), with the exceptions between *Stereognathus ooliticus* and both Russian species of *Stereognathus*, and between *Oligokyphus major* and *Dinnebitodon amarali*. *Stereognathus ooliticus* differs from the Russian Jurassic *Stereognathus* sp. in having significantly more numerous coarse scratches (CS) but does not differ significantly from the Cretaceous *Stereognathus sibiricus* in any of the four numeric parameters. The number of fine scratches (FS) differs significantly between eight of the 14 species pairs. The number of large pits (LP) only differs significantly between *Kayentatherium wellesi* and the Jurassic *Stereognathus* sp. with the latter having lower values. Three species pairs show the most possible, i.e. four, significant differences: *S*. *sibiricus*–*K*. *wellesi* and *S*. *sibiricus*–*D*. *amarali* (SP, FS, G, PP); *K*. *wellesi*–*Stereognathus* sp. (SP, LP, FS, G). There are no significant differences between the two Russian species of *Stereognathus*.

**Table 2:** Stereoscopic microwear parameters for all individuals.

Taxon and country collected	Collection ID	SP	LP	TP	FS	CS	TS	G	PP
*Oligokyphus* sp. (GB)	NHMUK PV R 37376	9.5	6.75	16.3	6.4	2	8.4	0	1
*Oligokyphus* sp. (US)	MCZ 111 AR 83	6.5	7.5	14	3.75	1	4.75	0	1
*Oligokyphus triserialis* (DE)	GPIT-1577-8	10.5	5.5	16	6	0.5	6.5	1	1
*Tritylodon longaevus* (ZA)	FMNH P30036	13	1	14	7.25	0	7.25	0	0
*Oligokyphus major* (GB)	BRSUG 26300	14.9	3.3	18.2	4.2	0.7	4.9	0	1
	BRSUG 26295_D	14.3	7.75	22	6.5	4	10.5	0	0
**Mean**		**14.6**	**5.53**	**20.1**	**5.35**	**2.35**	**7.7**	**0**	**0.5**
**St Dev**		**0.46**	**3.15**	**2.69**	**1.63**	**2.33**	**3.96**	**0**	**0.71**
*Stereognathus ooliticus* (GB)	GLRCM H38-9	10	2.5	12.5	2.5	0.13	2.63	0	0
	GLRCM 75710	9.25	6.5	15.8	7.75	1	8.75	0	0
	GLRCM 2104	9.75	6.25	16	8	5.25	13.3	0	0
	GLRCM H 174	11.7	4	15.7	5.67	4.33	10	0	0
	GLRCM TRAY-12-22	10	8.5	18.5	6.25	4.75	11	1	1
*Stereognathus* cf. *ooliticus* (GB)	FMNH P30037	10.5	6.5	17	8.5	6	12.5	0	0
**Mean**		**10.3**	**5.34**	**15.6**	**5.70**	**2.45**	**8.01**	**0.14**	**0.44**
**St Dev**		**3.70**	**2.21**	**4.48**	**2.04**	**2.04**	**3.23**	**0.36**	**0.48**
*Kayentatherium wellesi* (US)	MCZ 8837	24	7.5	31.5	17	0	17	0	0
	MCZ 8811	25	3.25	28.3	12.1	3.2	15.3	0	1
	USNM PAL 317208	24.8	8.25	33	11.8	2.5	14.3	0	0
**Mean**		**24.59**	**6.33**	**30.9**	**13.6**	**1.9**	**15.5**	**0**	**0.33**
**St Dev**		**0.52**	**2.7**	**2.43**	**2.93**	**1.68**	**1.39**	**0**	**0.58**
*Dinnebitodon amarali* (US)	MCZ 8830–1	13	4.75	17.8	18.3	5	23.3	0	1
	MCZ 8831	23.3	5	28.3	11	0	11	0	0
cf. *Dinnebitodon amarali* (US)	MCZ 4 (8931)	16.5	5.25	21.8	7.5	1.75	9.25	0	0
**Mean**		**17.6**	**5**	**22.6**	**12.3**	**2.25**	**14.5**	**0**	**0.33**
**St Dev**		**5.24**	**0.25**	**5.33**	**5.48**	**2.54**	**7.63**	**0**	**0.58**
*Stereognathus* sp. (RU)	ZIN PH 63/117	9	3	12	4	1.25	5.25	1	1
	ZIN PH 64/117	10	0	10	4.5	0	4.5	0	1
	ZIN PH 65/117	10	2.5	12.5	3	0	3	1	1
	ZIN PH 66/117	9.5	0	9.5	3	0	3	0	0
	ZIN PH 67/117	5.5	2	7.5	2.5	0	2.5	1	1
	ZIN PH 68/117	11	3	14	3.5	0	3.5	1	1
	ZIN PH 69/117	9	2	11	3.5	0	3.5	1	0
	ZIN PH 70/117	8.5	2	10.5	5	0	5	1	0
	ZIN PH 71/117	7.5	1	8.5	3.5	0	3.5	1	1
	ZIN PH 72/117	11.5	2	13.5	5.5	0	5.5	1	0
	ZIN PH 73/117	9	3.5	12.5	5.5	0.5	6	1	1
	ZIN PH 74/117	8	1	9	4	0	4	0	0
	ZIN PH 75/117	8.5	4.5	13	4.5	0	4.5	1	1
	ZIN PH 76/117	6.5	3	9.5	5	0	5	1	1
**Mean**		**8.82**	**2.11**	**10.9**	**4.07**	**0.13**	**4.20**	**0.79**	**0.64**
**St Dev**		**1.62**	**1.29**	**2.02**	**0.96**	**0.35**	**1.07**	**0.43**	**0.5**
*Stereognathus sibiricus* (RU)	ZIN PH 3/154	7.5	5	12.5	4.5	2	6.5	1	1
	ZIN PH 4/154	7	2.5	9.5	4.5	0	4.5	0	1
	ZIN PH 5/154	10	5	15	7.5	2.5	10	1	1
	ZIN PH 6/154	9.5	6	15.5	4.5	1.5	6	1	1
	ZIN PH 7/154	5.5	2	7.5	4	0.5	4.5	1	1
	ZIN PH 8/154	8	3	11	7.5	1	8.5	1	1
	ZIN PH 9/154	10	2.5	12.5	4	0	4	1	1
**Mean**		**8.21**	**3.71**	**11.9**	**5.21**	**1.07**	**6.29**	**0.86**	**1**
**St Dev**		**1.70**	**1.58**	**2.86**	**1.58**	**0.98**	**2.25**	**0.38**	**0**

Numbers show individual means, species means (Mean; bold), and standard deviations (St Dev; bold). Abbreviations (ordered as in table): SP = mean number of small pits; LP = mean number of large pits; TP = mean number of total pits; FS = mean number of fine scratches; CS = mean number of coarse scratches: TS = mean number of total scratches; G = gouges; PP = puncture pits; 0 = feature absent; 1 = feature present

**Table 3:** Pairwise comparisons of tritylodontid species with N ≥ 2.

	*Stereognathus ooliticus*	*Kayentatherium wellesi*	*Dinnebitodon amarali*	*Stereognathus* sp.	*Stereognathus sibiricus*
***Oligokyphus major***	SP	SP, FS, *Spc*	FS	SP, G	SP, G
***Stereognathus ooliticus***		SP, FS	SP, FS	CS, G	G, PP
***Kayentatherium wellesi***			SP	SP, LP, FS, G	SP, FS, G, PP
***Dinnebitodon amarali***				SP, FS, G, *Spd*	SP, FS, G, PP, *Spd*
***Stereognathus* sp.**					–

Pairwise comparisons depict significant differences (p<0.05) of SP (number of small pits), LP (number of large pits), FS (number of fine scratches), CS (number of coarse scratches), G (presence of gouges), PP (presence of puncture pits), *Spc* (curvature of the peaks), and *Spd* (peak density). Surface texture parameters are in italics.

A Kruskal-Wallis test for the two non-parametric parameters (presence/absence of gouges; presence/absence of puncture pits) was run for the individual means of the species with N≥2. The two Russian *Stereognathus* species similarly bear numerous gouges (G) but they are significantly different from all other species, which have no G or low presence values. The Cretaceous *Stereognathus sibiricus* is the only taxon having a 100% presence of puncture pits (PP) and hence differs significantly from *Stereognathus ooliticus*, *Kayentatherium wellesi*, and *Dinnebitodon amarali*.

Species with N = 1 [*Oligokyphus* sp. (GB), *Oligokyphus* sp. (US), *Oligokyphus triserialis*, *Tritylodon longaevus*] could not be tested statistically. Regarding SP, all have low or low to moderate values, which are very different from *Oligokyphus major*, *Kayentatherium wellesi*, and *Dinnebitodon amarali*. *Oligokyphus* sp. (US) has the highest and *Oligokyphus* sp. (GB) the second highest values of LP and, therefore, are very different from *Tritylodon longaevus* and both Russian *Stereognathus* species. *Tritylodon longaevus* has the lowest number of large pits, i.e. a single large pit, and lacks CS, gouges (G), and puncture pits (PP), and is thus the taxon with the fewest coarse features. The small-bodied *Oligokyphus* sp. (US) has the lowest number of FS (n = 3.75) and in this respect is similar to *O*. *major*, and the two Russian *Stereognathus* species (the last two medium-bodied) but different from *K*. *wellesi*, *D*. *amarali*, and *T*. *longaevus*. *Oligokyphus triserialis* has a very low value for CS (n = 0.5) but features both G and PP and is thus similar to both Russian *Stereognathus* species. *Oligokyphus* sp. (GB) and *Oligokyphus* sp. (US) both lack G and, therefore, are different from the Russian *Stereognathus* species, but show PP like the latter and *O*. *triserialis*.

**Fig 3:**
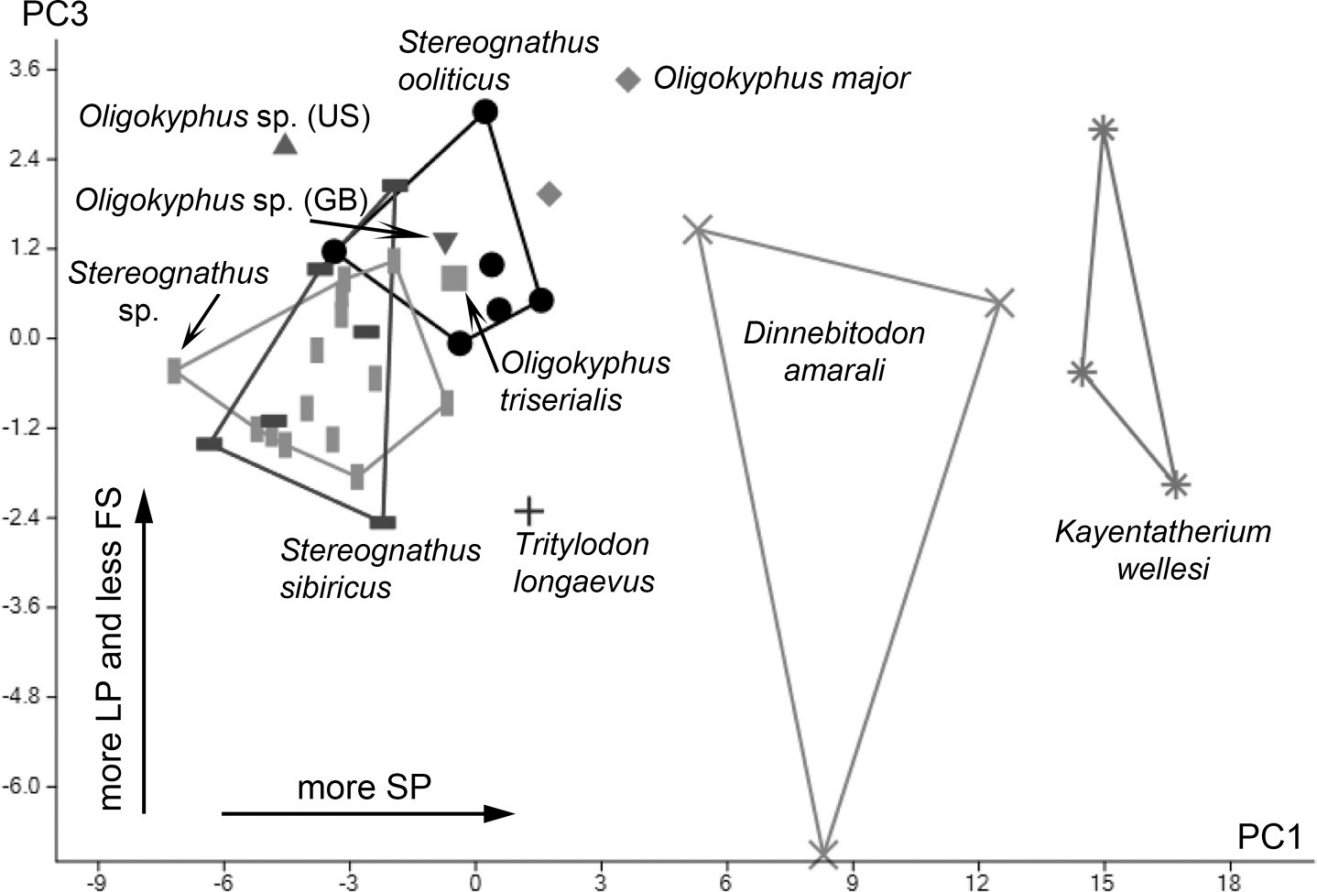
Scatter plot of principle components 1 and 3 on stereoscopic microwear parameters. Stereoscopic microwear parameters used are: number of small pits (SP); number of large pits (LP); number of fine scratches (FS); number of coarse scratches (CS). Convex hulls embrace areas taken by taxa with N > 2; symbols represent individual means.

A principal component analysis (PCA) (Fig 3, S3 Table) was run on a variance-covariance matrix including individual mean values of the following four numerical wear parameters showing significant differences: number of small pits; number of large pits; number of fine scratches; number of large scratches. The first principal component (PC1) is strong and explains about 74.5% of the total variance, the second component (PC2) explains about 15%, and the third component (PC3) about 7.5%. We, therefore, considered components beyond the third to be insignificant in terms of their contribution to the total variance, and excluded these from further analyses.

PC1 features a high positive loading for the number of small pits. *Kayentatherium wellesi* and *Dinnebitodon amarali* plot furthest right on the scatter plot with the highest small pit values, followed by *Oligokyphus major* with somewhat lower values. *Oligokyphus* sp. (US) and the two Russian species of *Stereognathus* have the lowest small pit values and plot at the left end of the scatter plot. PC2 shows character loadings only below the cut-off of 0.7; it is most influenced by the number of large pits. PC3 shows a high negative loading above the cut-off for the number of fine scratches. With the highest values, *D*. *amarali*, *Kayentatherium wellesi* and the single tooth of *Tritylodon longaevus* tend towards the lower end of the scatter plot. *Oligokyphus* sp. (US) and *O*. *major* can be found at the upper end of the plot.

**Fig 4:**
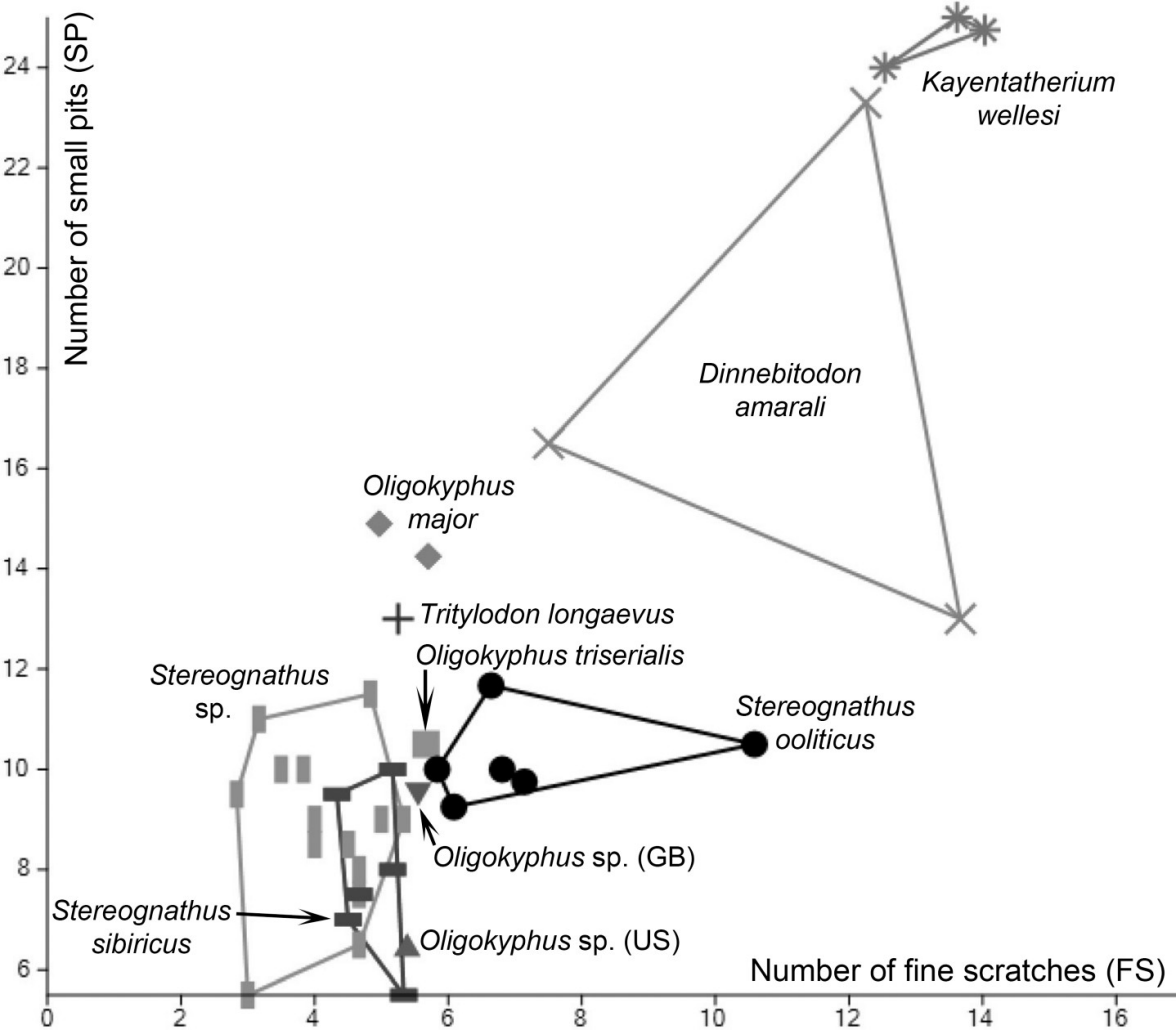
Bivariate plot of stereomicroscopic microwear parameters: Number of fine scratches (FS) versus number of small pits (SP). Convex hulls embrace the areas taken by taxa with N > 2; symbols represent individual means.

The bivariate plot (Fig 4) compares the two principal components (number of fine scratches versus number of small pits) for individual mean values of all ten taxa analyzed. *Kayentatherium wellesi* shows values well above ten for the number of FS and well above 20 for the number of SP and, therefore, plots at the uppermost right corner of the field. Values for *Dinnebitodon amarali* are variable but still high for both characters. *Oligokyphus major* and *Tritylodon longaevus* have values well above 10 for the number of SP but lower values for the number of FS. *Stereognathus ooliticus* tends towards having higher values for the number of FS but most individuals have values around seven and around ten for SP. Both Russian *Stereognathus* species have low values for the number of FS and the number of SP and, therefore, plot at the lowermost left corner of the scatter plot. However, *Stereognathus sibiricus* tends to have somewhat higher values of FS compared with the Jurassic species of Russian *Stereognathus*. *Oligokyphus triserialis* and *Oligokyphus* sp. (GB) plot in between the areas occupied by *S*. *ooliticus* and the space filled by the two Russian *Stereognathus* species. *Oligokyphus* sp. (US) is characterized by very low values for the number of SP and, therefore, plots near the bottom of the graph.

### 3D surface texture

One-way ANOVA results (species with N≥2) show significant differences in peak curvature (*Spc*) and peak density (*Spd*) between three of the 14 species pairs only (Table 3). The absence of significant differences in mean dale area (*Sda*) and pit void volume (*Vvv*) indicates that all species overlap and are very similar (Table 3, S2 Table). However, the Tukey’s pairwise comparison reveals that *Kayentatherium wellesi* has sharper peaks (smaller peak curvature *Spc*) than *Oligokyphus major* from GB. Both Russian taxa of *Stereognathus* have smaller peak densities (*Spd*) than the North American taxon *Dinnebitodon amarali* (Table 3).

Species with N = 1 [*Oligokyphus* sp. (GB), *Oligokyphus* sp. (US), *Oligokyphus triserialis*, *Tritylodon longaevus*] could not be tested statistically. Regarding peak density (*Spd*), *Oligokyphus* sp. from US has the largest values of all species; *Oligokyphus triserialis* is closest to *Oligokyphus* sp. (GB) and *Tritylodon longaevus* overlaps with the three *Stereognathus* taxa (Table 4).

**Table 4:** Surface texture parameters for all individuals.

Taxon and country collected	Collection ID	*Vvv*	*Spd*	*Spc*	*Sda*
*Oligokyphus* sp. (GB)	NHMUK PV R 37376	0.216	0.021	47.01	15.98
*Oligokyphus* sp. (US)	MCZ 111 AR 83	0.458	0.063	63.25	13.08
*Oligokyphus triserialis* (DE)	GPIT-1577-8	0.142	0.009	237.34	132.71
*Tritylodon longaevus* (ZA)	FMNH P30036	0.221	0.002	133.26	86.49
*Oligokyphus major* (GB)	BRSUG 26300	0.124	0.004	97.56	99.78
	BRSUG 26295_D	0.168	0.014	168.36	47.71
**Mean**		**0.221**	**0.019**	**124.46**	**65.96**
**St Dev**		**0.122**	**0.023**	**71.01**	**48.27**
*Stereognathus ooliticus* (GB)	GLRCM H38-9	0.140	0.010	61.23	38.84
	GLRCM 75710	0.310	0.022	69.01	34.94
	GLRCM 2104	0.103	0.002	80.89	265.76
	GLRCM H 174	0.080	0.001	45.90	74.50
	GLRCM TRAY-12-22	0.066	0.001	43.25	38.41
*Stereognathus* cf. *ooliticus* (GB)	FMNH P30037	0.113	0.001	101.24	90.72
**Mean**		**0.135**	**0.006**	**66.92**	**90.53**
**St Dev**		**0.089**	**0.009**	**21.96**	**88.81**
*Kayentatherium wellesi* (US)	MCZ 8837	0.103	0.002	20.57	163.35
	MCZ 8811	0.101	0.010	35.43	100.88
	USNM PAL 317208	0.070	0.010	24.88	51.82
**Mean**		**0.091**	**0.007**	**26.96**	**105.35**
**St Dev**		**0.018**	**0.005**	**7.65**	**55.90**
*Dinnebitodon amarali* (US)	MCZ 8830–1	0.074	0.011	9.51	69.35
	MCZ 8831	0.097	0.008	24.52	89.52
cf. *Dinnebitodon amarali* (US)	MCZ 4 (8931)	0.106	0.029	63.55	29.29
**Mean**		**0.092**	**0.016**	**32.53**	**31.36**
**St Dev**		**0.017**	**0.012**	**27.90**	**27.42**
*Stereognathus* sp. (RU)	ZIN PH 63/117	0.232	0.001	45.74	40.92
	ZIN PH 64/117	0.114	0.001	62.44	11.69
	ZIN PH 65/117	0.137	0.003	56.56	245.73
	ZIN PH 66/117	0.147	0.002	47.53	105.22
	ZIN PH 67/117	0.185	0.001	48.03	220.94
	ZIN PH 68/117	0.182	0.001	32.64	44.02
	ZIN PH 69/117	0.109	0.002	20.22	152.38
	ZIN PH 70/117	0.129	0.004	65.15	373.40
	ZIN PH 71/117	0.108	0.002	12.07	289.59
	ZIN PH 72/117	0.132	0.004	118.07	59.22
	ZIN PH 73/117	0.097	0.001	12.45	156.77
	ZIN PH 74/117	0.192	0.001	48.69	211.98
	ZIN PH 75/117	0.067	0.002	1.40	339.89
	ZIN PH 76/117	0.118	0.001	62.35	144.21
**Mean**		**0.139**	**0.002**	**45.24**	**171.14**
**St Dev**		**0.044**	**0.001**	**29.51**	**114.29**
*Stereognathus sibiricus* (RU)	ZIN PH 3/154	0.067	0.001	14.85	195.29
	ZIN PH 4/154	0.146	0.002	124.22	356.25
	ZIN PH 5/154	0.272	0.002	93.91	11.67
	ZIN PH 6/154	0.064	0.001	8.66	634.19
	ZIN PH 7/154	0.085	0.003	1.39	238.98
	ZIN PH 8/154	0.129	0.003	54.79	152.07
	ZIN PH 9/154	0.117	0.002	81.48	408.86
**Mean**		**0.126**	**0.002**	**54.19**	**285.33**
**St Dev**		**0.072**	**0.001**	**47.67**	**202.03**

Numbers show individual means, species means (Mean; bold), and standard deviations (St Dev; bold). Abbreviations (ordered as in table): *Vvv* = pit void volume (μm^3^/μm^2^); *Spd* = peak density (1/μm^2^), *Spc* = arithmetic mean peak curvature (1/μm); *Sda* = mean dale area (μm^2^).

**Fig 5:**
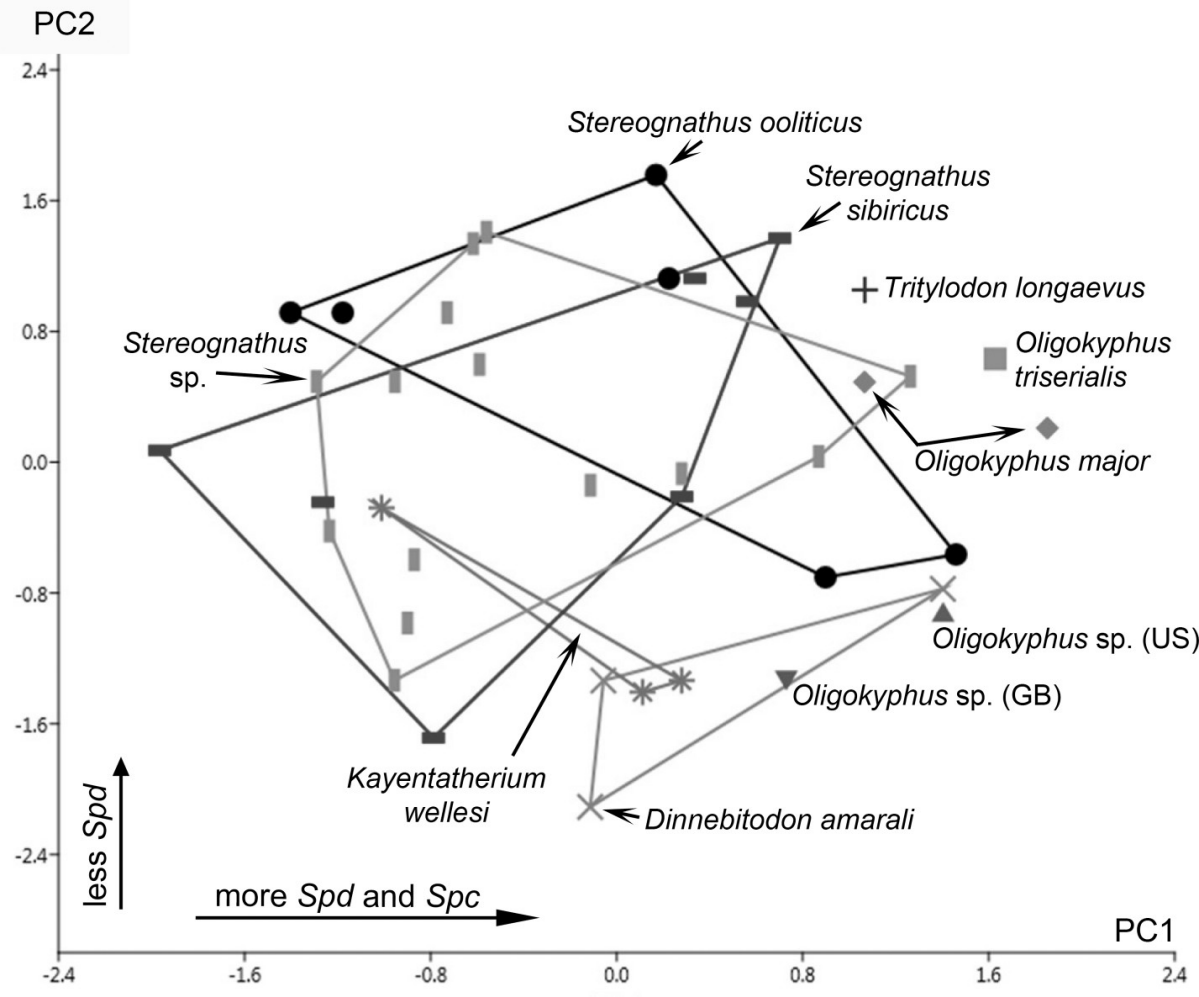
Scatter plot of principle components 1 and 2 on 3D surface texture parameters. 3D surface texture parameters used are: pit void volume (*Vvv* in μm^3^/μm^2^), mean dale area (*Sda* in μm^2^), peak density (*Spd*, 1/μm^2^) and arithmetic mean peak curvature (*Spc*, 1/μm). Convex hulls embrace the areas taken by each taxon (for taxa with n > 2); symbols represent individual means.

A principle component analysis (PCA) was run on a variance-covariance matrix including individual means of the four numerical and transformed parameters (rank-transformed for *Spc* and *Spd* and log-transformed for *Vvv* and *Sda*). The first component explains about 61% of the total variance, the second component explains about 38.9%, and the third component about 0.07%; loadings are given in the S3 Table and the scatter plot of component 1 and 2 in Fig 5. PC1 features the highest positive loadings for peak curvature and peak density, whereas void volume and dale area are comparably very low. The main loadings are not particularly high and only very slightly above the cut-off of 0.7. On PC2, peak density has a negative and peak curvature a positive loading explaining most of the vertical spread of the data. The three *Stereognathus* taxa are in a centric position and overlap most, whereas *Dinnebitodon* plots in the most distinctive position in the right lower part with only slight overlap with *Kayentatherium*. Most *Oligokyphus* individual mean data points are on the right of PC1, as is that of *Tritylodon triserialis*.

**Fig 6:**
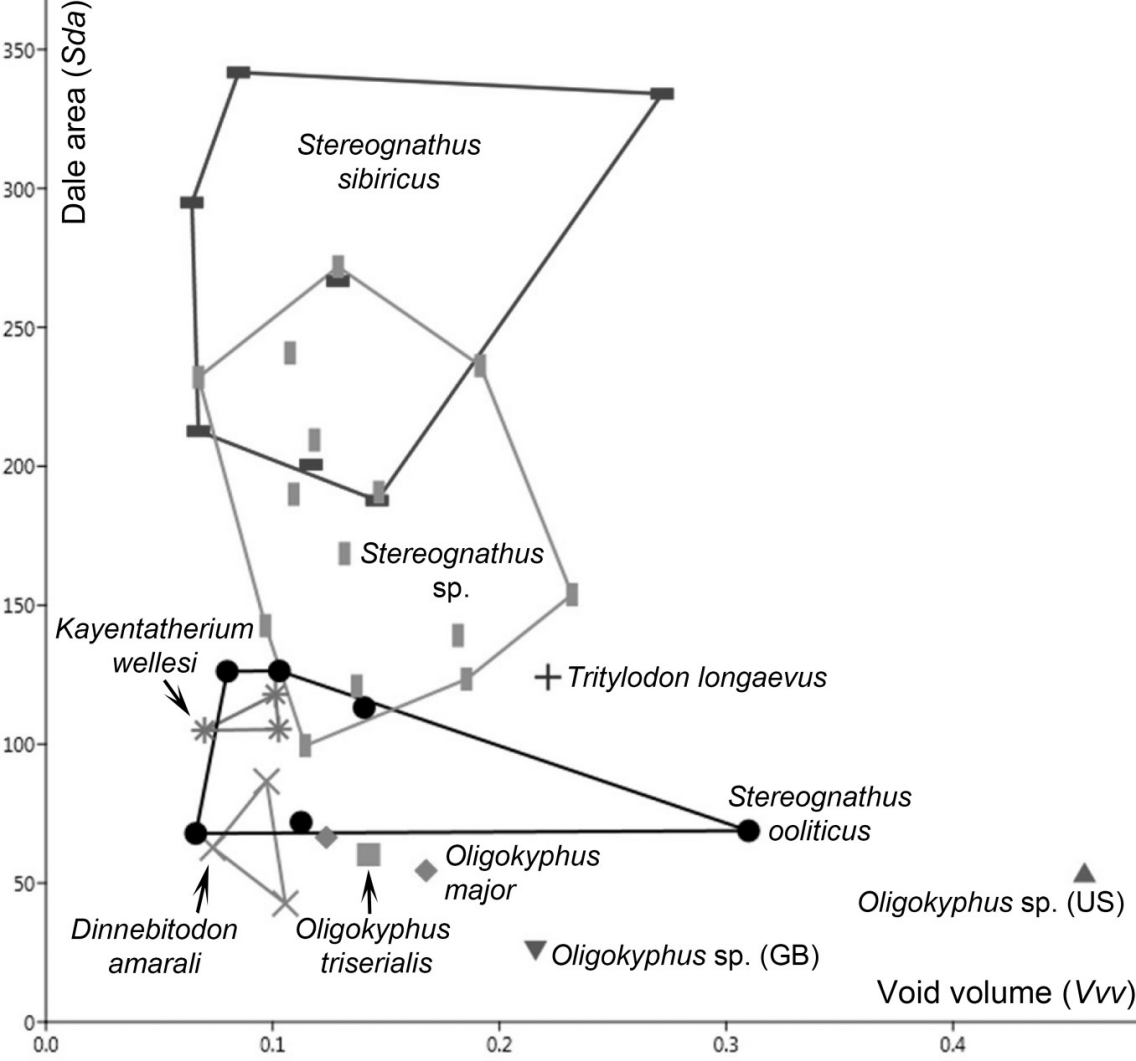
Bivariate plot of surface texture parameters pit void volume (*Vvv* in μm^3^/μm^2^) versus mean dale area (*Sda* in μm^2^). Convex hulls embrace the area taken by each taxon (for taxa > 2); symbols represent individual means.

The bivariate plot (Fig 6) is made with the individual mean values for the parameters pit void volume (*Vvv* in μm^3^/μm^2^) and mean dale area (*Sda* in μm^2^) of all ten taxa analyzed. As indicated by ANOVA and PCA, a large overlap between the three *Stereognathus* taxa is evident denoted by a narrow range of void volume (*Vvv*) and the widest variety of dale area (*Sda*) for both taxa from Russia. However, *Stereognathus ooliticus* from GB has the lowest *Sda* values of the three *Stereognathus* taxa, but also has a larger variety of dale area (*Sda*) overlapping in that parameter with *Dinnebitodon* and *Kayentatherium*.

## Discussion

Both approaches (stereomicroscopic microwear and 3D surface texture) independently determined that most taxa were omnivores with heterogeneous plant parts and animal matter, probably mostly insects, as major food resources. We do not see any signs of the exploitation of roots, tubers, or other underground repository organs of plants, an adaptation that primarily produces extensive coarse and hypercoarse microwear features (pits, scratches, and gouges) on cheek teeth in mammals [45]. However, feeding on subaerial organs, such as hard-seeds and cones can produce large pits, puncture pits, or coarse scratches as found on the analyzed tooth surfaces [48]. The rounded and less frequent peaks in surface texture features are interpreted as evidence for little abrasion by extrinsic mineral grains [49–51]. We link the rounding of the peaks to attritional contacts and polishing by fluid-rich materials [51], such as softer plant components (e.g., fleshy seed coats, seed kernels, leaf mesophyll, fleshy megasporophylls, or arils) or animal components (e.g., insect viscera enclosed in the exoskeleton).

We are aware of the varied problems when comparing tritylodontid dental microwear and 3D surface texture features with those in extant mammals. Using extant mammals as a comparative dataset is beneficial because most published results on stereomicroscopic microwear/3D surface textures of teeth are based on studies of modern mammals (comprising, to a large extent, ungulates and primates [45, 48, 50, 52–54], but also small mammals [49, 55, 56]). However, such comparisons are problematic because of the floral disparity between geological periods (gymnosperms dominated Mesozoic floras, whereas angiosperms dominated Cenozoic vegetation). Further, the backward-directed power stroke during the chewing movement of tritylodontids (to our knowledge not employed by any extant mammal) and the equivocal comparability of life styles and habitats between tritylodontids and modern mammals make direct comparisons of feeding behavior difficult. Only two studies to date have analyzed dental microwear features in non-mammalian cynodonts: Goswami et al. [57] investigated Traversodontidae using scanning electron microscopy; Kubo et al. [58] analysed the dental microwear of *Exaeretodon argentinus* (Therapsida) using confocal microscopy. In the following section, we use the extant mammal dataset for comparisons, but we stress that our interpretations are with caution and discretion.

### Stereomicroscopic microwear

We observed a predominantly fine-wear signature in all ten analyzed taxa, suggesting an overall low to moderate abrasiveness of food items. The one-way ANOVA and PCA identified the number of small pits (SP) and number of fine scratches (FS) as the most powerful parameters segregating taxa (Table 3); SP numbers always exceed the numbers of FS. However, almost all microwear profiles of the tritylodontids tested reflect the feeding on hard food items or exogenous effects (contamination of food with grit), or both to some degree.

None of the analyzed taxa within Tritylodontidae was a dietary specialist on the basis of the stereoscopic microwear results. All species have plant parts in their diet, in combination with soft- and/or hard-bodied insects, the latter having a moderate abrasive effect on tooth surfaces [59–61]. However, *Tritylodon longaevus* and the two Russian *Stereognathus* species feature only low to moderate counts of pits and scratches of all kinds suggesting that insects were a food resource of minor importance. Hard objects, such as leathery cones/megasporophylls and/or seeds were regularly consumed by the *Oligokyphus* taxa tested and by the two Russian *Stereognathus*.

The large-bodied *Kayentatherium wellesi* and the medium-bodied *Dinnebitodon amarali* are similar in the microwear profiles of the individual means (Fig 4, Table 3). Both have high frequencies of SP, LP, and FS; moderate frequencies of CS; and low frequencies of G and PP. However, *K*. *wellesi* features significantly more SP than *D*. *amarali*. We correlate both profiles with the consumption of food items of moderate intrinsic abrasiveness without the noticeable influence of extrinsic factors (no gouges). High counts of SP, as in *Kayentatherium* and *Dinnebitodon*, were for example observed in the muroid rodent *Acomys spinosissimus* [55], which is known to feed on both seeds and insects [62]. A high frequency of pits of all sizes was noticed in other species with an insectivore diet [59]. Therefore, we suggest that the main food sources of both taxa (and especially *K*. *wellesi*) were softer seeds, arils, or similar parts (on the basis of low PP counts) and soft- and/or hard-bodied insects. Owing to the high frequency of microwear features, we interpret subaerial plant parts of heterogeneous anatomy, such as leaves and shoots to have been subsidiary food resources.

The microwear profile of the single tooth of the large-bodied *Tritylodon longaevus* shows moderate frequencies of SP and FS; all other parameters have low frequencies (Fig 4, Table 3). This suggests the intake of food items of low to moderate intrinsic toughness without the noticeable influence of extrinsic factors. Possible food items include subaerial plant parts of heterogeneous anatomy including leaves, shoots, and plant reproductive organs. Occasional consumption of animal matter might have occurred.

The microwear profiles of the two Russian *Stereognathus* taxa (Fig 4, Table 3) are more similar to each other than either are to *Stereognathus ooliticus* (GB) (all medium-bodied). *Stereognathus sibiricus* and *Stereognathus* sp. have low or low to moderate numbers of SP and CS, and LP and FS, respectively, which we relate to the intake of soft to moderately tough food items (subaerial plant parts of heterogeneous anatomy). In contrast, the moderate to high presence of PP in both Russian taxa, especially in the Cretaceous *S*. *sibiricus*, points to the regular consumption of thick-walled seeds, cones, or other hard to leathery plant structures. The high abundance of G suggests the influence of extrinsic factors that might include contaminants of food, such as grit from the acquisition of food close to the ground. Occasional consumption of animal matter might also have occurred.

*Stereognathus ooliticus* (GB) shows moderate to high abundances of SP, LP, FS, and CS, and in contrast, very low representation of G and PP (Fig 4, Table 3). Interestingly, it is the only species having high counts of both coarse features LP and CS and, regarding the latter, it is significantly different from the Russian *Stereognathus* sp. This microwear profile correlates with a mixed diet of plants and insects. It suggests consumption of food items with moderate to high intrinsic toughness, but no substantial seed or cone herbivory and negligible influence of extrinsic factors (only one of six specimens showed PP and G). Possible food items include heterogeneous plant parts with tough coatings/bark (i.e., with many cell layers thickened and strengthened by lignin or suberin) resulting in high numbers of CS. A high number of LP is evident in species that eat high quantities of insects [59].

All four species of *Oligokyphus* are small-bodied and their microwear profiles are rather similar: low to moderate numbers of SP, FS and CS, a high number of LP, and a moderate to high presence of PP (Fig 4, Table 3). In contrast, no G were recorded except in the one specimen of *O*. *triserialis*. This solitary occurrence is interpreted to signal grit/dust intake in a somewhat drier habitat. The profiles suggest an opportunistic diet, including items with low to moderate intrinsic toughness; the high LP counts and the high presence of PP accounts for regular predation on insects and/or consumption of plant reproductive structures including seeds.

### 3D surface texture

In the 3D surface texture analysis, all analyzed taxa have relatively low values of dale area (*Sda*), medium peak curvature (*Spc*) and low peak density (*Spd*), but with some deep pit voids as indicated by large values of pit void volume (*Vvv*) (Fig 5, Table 4). In general, the small–medium void depths and small–medium dale areas favor an intermediate diet of soft food items with some small but abrasive components [51], which we link to hard seed coats, for all taxa (Fig 6). The three *Stereognathus* taxa have a small–medium range of peak density (*Spd*), peak curvature (*Spc*) and void volume (*Vvv*), which suggests that they all must have had a similar diet. Comparable to the results of the stereoscopic microwear analysis, the surface texture data revealed a similar intermediate diet including both insects and plant parts. Since there is no comparative data from extant representatives, we base our interpretations on functional analogies following the ingestion and mastication related hypotheses [51]. In accordance with [51], *Dinnebitodon* and some of the *Kayentatherium* specimens also have very fine and shallow voids and are interpreted to be candidates for consumption of seeds with low-durability coats. The fine and less deep textural features of all taxa analysed further suggest low chewing forces and relatively few degrees of freedom for the chewing movements, which is further supported at a larger scale by the steep flanks and high cusp tips of teeth.

Comparing our surface texture results with surface texture-based dietary reconstructions proposed for earliest stem mammals [63], or small-bodied insectivorous mammalian bats [64], is technically challenging and only broad inferences can be drawn. Data available for both groups has been acquired applying a method called focus variation at lower resolutions, using different instrumentation, preprocessing data filtering routines and parameter sets. The only parameter used herein corresponding to those studies is pit void volume, which we link to plant as well as insect hard objects in the diet; and not only to insect prey ‘hardness’ as used by Purnell et al. [64] and Gill et al. [63]. However, for the tritylodontid sample, the peak density and peak curvature seem to be more important, but were not considered in the analyses by Gill et al. [63] and Purnell et al. [64].

### Possible candidates for plants in the diet

Unless tritylodontids were extremely specialized in their feeding strategies, it is likely that the non-animal component of their diets derived from one or more of the relatively common plant groups and plant organs of the Mesozoic. On this basis, we exclude rare and geographically or stratigraphically restricted plant groups (e.g., Petriellales, *Sanmiguelia*, *Furcula*, *Anthrophyopsis*, Iraniales) from these analyses.

Given their range of dental wear features, we infer that tritylodontids consumed a mix of soft, moderate and hard plant components but relatively small amounts of mineral matter, thus excluding roots, rhizomes and tubers as primary and/or secondary food resources. Major plant groups available for herbivory did not vary greatly between the Late Triassic and Early Cretaceous (S4 Table). After the Aptian, angiosperms became significant components of the global floras [65]. However, since the youngest tritylodontids in this study are of Aptian or greater age, we exclude angiosperms as significant components of their diet for most of their history. Within each plant group, different organs and tissue types likely induced varying degrees of abrasion on vertebrate teeth (Table 5).

**Table 5:** Interpreted hardness of specific plant tissues as components of vertebrate diets.

Tissue type	Main distribution in plants	Presence in seeds, cones and other reproductive structures	Relative hardness
Xylem (tracheids and vessels)	Generally thick in stems and forming major component of leaf veins of gymnosperms; forming strands in variable arrangement in free-sporing plants	Prominent in cone axes and bracts, minor in seeds	Moderate to hard owing to lignin thickening over cellulose
Cambium and phloem	Sparse in free-sporing plants	Minor	Soft, thin, cellulose-walled cells
Parenchyma	Abundant in leaves, pith and roots of woody plants and in cortex of herbaceous stems. Also occur in enlarged form in specialized starch-storage organs	Locally abundant in seeds with fleshy coats or supported by fleshy arils or receptacles	Soft, thin, cellulose-walled cells; but in some cases associated with hard siliceous phytoliths
Sclerenchyma (sclerieds and fibres)	Scattered in stems, leaves and reproductive structures as support and defensive structures	Locally abundant in seeds with hard coats	Hard-walled cells of cellulose and hemicellulose coated by lignin
Collenchyma	Mostly in angiosperms but potentially in many extinct seed ferns, especially adjacent to vascular bundles	Minor	Moderate hardness owing to irregularly thickened walls of cellulose and pectin
Periderm	Forming secondary tissues of the outer bark of woody plants	Absent?	Moderate to hard tissue owing to thickening of cell walls by suberin
Epidermis and hypodermis	Forming cell layers adjacent to the surface of leaves and young stems	Present: one- to several-layered	Low to moderate hardness; usually associated with waxy cuticle
Gametophyte and embryo tissues	Within seeds of gymnosperms; forming a diminutive separate generation in free-sporing plants	Inner ‘kernel’ part of seeds	Soft—mostly parenchymatous storage cells.

With respect to body size, small mammalian herbivores tend to be more selective feeders on high-reward items to maintain high metabolic rates. On this basis, we infer that small-bodied tritylodontids (e.g., the four species of *Oligokyphus* analysed here) also very likely favoured plant components with higher nutritional values per unit-mass of consumed tissue, such as seeds, other reproductive organs, shoot tips or specialized starch-reserve storage structures (e.g., bulbils, corms/rhizomorphs). These organs have a mix of hard (e.g., sclerenchymatous seed coat) moderate (e.g., fibre bundle) and soft (e.g., parenchyma) tissues that are consistent with the mixed sets of abrasion features evident on the teeth of *Oligokyphus*. In the absence of preserved gut contents or coprolites, we are unable to definitively identify plant groups targeted by tritylodontids. However, on the basis of dental wear, plant stature and distribution, representation of tissue types, and Mesozoic evidence for endochory in some plant groups [66], we suggest that seeds/cones of conifers and Gnetales, and the ovuliferous reproductive structures and dispersed seeds of Ginkgoales, Cycadales and Mesozoic seed ferns were prime potential food sources for Tritylodontidae.

By the mid-Mesozoic, most modern hexapod groups, with the exception of social insects, were established and represented potential prey of tritylodontids [67]. Chitinous exoskeletons of adult insects are known to have a moderate abrasive effect as food items [60, 61]. Insect larvae, pupae, nymphs, and naiads are somewhat softer prey items owing to their unmineralised thinner exoskeletons. Other terrestrial invertebrates, especially other terrestrial and freshwater arthropods and/or annelids constituted additional potential prey items of widely variable hardness. Fungal fruiting bodies with soft to moderate-hardness chitin-walled cells may have represented a further source of nutrition.

Recently, a semiaquatic lifestyle has been proposed for *Kayentatherium wellesi* [68]. However, there appear to have been few aquatic vascular plants prior to the Early Cretaceous apart from small cormose isoetalean lycophytes and emergent reed-like equisetaleans. In the early part of the Cretaceous, and especially later in that period, a revolution in freshwater ecosystems was triggered by the rapid diversification of aquatic angiosperms, water-ferns and herbaceous isoetaleans [69, 70]. The dominant primary producers in Triassic to Early Cretaceous freshwater ecosystems were probably charophytes, other macroalgae and planktonic algae. Feeding on freshwater algae seems unlikely for tritylodontids since they are of low nutritional value. An aquatic lifestyle might, however, signify a diet rich in freshwater invertebrates. Living and feeding in wetlands and/or close to water could explain the lack of gouges on tooth facets, but the latter feature is also expressed in five other analyzed tritylodont species of unknown lifestyle. It should be noted that in the two Russian *Stereognathus* species and the single tooth of *Oligokyphus triserialis*, a relatively high presence of gouges is evident, which can be interpreted as signal of increased occurrence of abiotic abrasives (‘grit‘) on food items in dry and dusty habitats.

### Influence of body size on food choice?

The tritylodontids used in this study comprise taxa of small (all four *Oligokyphus* species), medium (all three *Stereognathus* species, *Dinnebitodon amarali*), and large body size (*Tritylodon longaevus*, *Kayentatherium wellesi*). Whether food choice is influenced by different body size is discussed here.

Neither method produced a clear signal in support for hypothesis 2. Based on the results, small pits (SP) and fine scratches (FS) are the most powerful parameters to discriminate between taxa. We see identical combinations of these two features in small- and medium-bodied taxa, small-, medium-, and large-bodied taxa, and between medium- and large-bodied taxa. Similarly, no influence of body size was found in the surface texture results.

## Conclusions

As a novel approach, we applied two methods of tooth wear analysis (stereoscopic microwear and 3D surface texture analysis) to a sample of ten species of Tritylodontidae. Independently; both approaches detected dietary adaptations that differed between species. The comparative interpretation of the results from each method led to the following conclusions:

1. Hypothesis 1 (“All analyzed tritylodontid taxa subsisted on a predominantly herbivorous diet, i.e., primary and secondary food resources consist of living plants and/or shed plant parts”) is rejected. Both tooth wear approaches suggest a varied diet of plants and plant parts together with variable amounts of animal matter. We interpret that all ten tritylodontid taxa were opportunistic in their food choice.
2. Hypothesis 2 (“Body size had an influence on food choice”) is also rejected. Both tooth wear approaches were unable to detect a correlation of body size and food choice.
3. Very low importance of exogenous effects was found for both tooth wear approaches, i.e., dust/grit contamination of food items.
4. The predominately fine wear features and low values of surface texture parameters make foraging for subterranean food items highly unlikely. If, however, food was harvested from underground sources, tritylodontids probably used their rodent-like incisors to break up the food item for reaching the softer inner parts.
5. The two tooth wear approaches are in conflict regarding the three species of *Stereognathus*: features are similar in all three species for 3D surface texture; however, stereoscopic microwear found contradicting features for *Stereognathus* sp. and *S*. *sibiricus* on one hand (consumption of soft to moderately tough food items), and *S*. *ooliticus* on the other hand (consumption of moderate to highly tough food items).
6. We summarize primary and secondary food resources in the analyzed taxa as follows:
  - *Kayentatherium wellesi*, *Dinnebitodon amarali*: Food items of moderate intrinsic toughness (mixed diet of softer seeds and soft- and/or hard-bodied insects)
  - *Stereognathus* sp. and *Tritylodon longaevus*: Food items of low to moderate intrinsic toughness (mixed diet of subaerial plant parts of heterogeneous anatomy and softer seeds); insects as a subsidiary food resource
  - *Stereognathus sibiricus*: Food items of low to moderate intrinsic toughness (mixed diet of seeds, cones, or other hard to leathery plant structures); insects as a subsidiary food resource; some influence of extrinsic factors
  - *Stereognathus ooliticus*: Food items of moderate to high intrinsic toughness (mixed diet of subaerial plant parts with tough coatings/bark and soft- and/or hard-bodied insects); note that this interpretation is based only on the results from stereoscopic microwear
  - *Oligokyphus major*, *Oligokyphus* sp. (GB), *Oligokyphus* sp. (US), *Oligokyphus triserialis*: Food items of low to moderate intrinsic toughness (mixed diet of plant reproductive structures including seeds and soft- and/or hard-bodied insects); but some influence of extrinsic factors in *O*. *triserialis*
7. The application of two independent methods of tooth wear analysis—differing with respect to magnification, parameters quantified and illumination—strengthens the results. These complementary methods facilitate a more detailed dietary reconstruction and functional interpretation for Tritylodontidae.

## Supporting information

S1 Fig Examples of tooth morphology and wear facets in Tritylodontidae. (a) Skull of *Oligokyphus* sp. (MCZ 8843) in ventral view. This specimen was excluded from analysis because of postmortem alterations on the wear facets. (b) and (c) Wear facets (on high-resolution casts) of lower postcanines of *Kayentatherium wellesi* (MCZ 8811). In all three images, anterior is to the right.
(TIF)

S1 Table Specimen details of the tritylodontid taxa analysed. Country codes refer to ISO 3166–1 alpha-2. Abbreviations: L, large-bodied; M, medium-bodied; pc, lower postcanine; PC, upper postcanine; S, small-bodied. Acronyms to museum collections: BRSUG: Geology Museum, University of Bristol, Bristol, GB; FMNH: Finnish Museum of Natural History, Helsinki, Finland; GLRCM: Gloucester City Museum, Gloucester, GB; GPIT: Paläontologische Sammlung, Fachbereich Geowissenschaften, Universität Tübingen, DE; MCZ: Museum of Comparative Zoology, Harvard, Massachusetts, USA; NHMUK: Natural History Museum, London, GB; USNM PAL: National Museum of Natural History, Department of Paleobiology, Washington D.C., USA; ZIN PH: Paleoherpetological collection of the Zoological Institute, Russian Academy of Sciences, Saint Petersburg, RU.
(PDF)

S2 Table ANOVA test results of tritylodontid species with N ≥ 2. Numerical parameters of stereoscopic microwear (small pits, large pits, fine scratches, and coarse scratches) and parameters of 3D surface texture (pit void volume, peak density, peak curvature, and dale area) are shown. Significant p-values are in bold.
(PDF)

S3 Table PCA variances and loadings of the significant parameter variables for stereoscopic microwear and 3D surface texture. Abbreviations (as ordered in table): SP = small pits; LP = large pits; FS = fine scratches; CS = coarse scratches; *Spd*_rank = peak density (rank-transformed); *Spc*_rank = arithmetic mean peak curvature (rank-transformed); *Sda*_log = mean dale area (log-transformed); *Vvv*_log = pit void volume (log-transformed).
(PDF)

S4 Table Key characters of major, widely distributed, Mesozoic plant groups. (PDF)

## References

[1] Osborn HF. The reptilian subclasses Diapsida and Synapsida and the early history of the Diaptosauria. Mem Am Mus Nat Hist. 1903;1:449–507.

[2] Rowe TB. Definition, diagnosis and origin of Mammalia. J Vertebr Paleontol. 1988;8:241–264.

[3] Cope ED. The Tertiary Marsupialia. Am Nat. 1884;18:686–697.

[4] Fedak TJ, Sues H-D, Olsen PE. First record of the tritylodontid cynodont *Oligokyphus* and cynodont postcranial bones from the McCoy Brook Formation of Nova Scotia, Canada. Can J Earth Sci. 2015;52(4):244–249.

[5] Hennig E. Die Säugerzähne des württembergischen Rhät-Lias-Bonebeds. N Jb Min Geol Paläont, Beil. 1922;46:181–267.

[6] Clemens WA, Martin T. Review of the non-tritylodontid synapsids from bone beds in the Rhaetic Sandstone, southern Germany. Paläont Z. 2014;88(4):461–479.

[7] Matsuoka H, Kusuhashi N, Corfe IJ. A new Early Cretaceous tritylodontid (Synapsida, Cynodontia, Mammaliamorpha) from the Kuwajima Formation (Tetori Group) of central Japan. J Vertebr Paleontol. 2016;36(4):e1112289.

[8] Tatarinov LP, Matchenko EN. A find of an aberrant tritylodont (Reptilia, Cynodontia) in the Lower Cretaceous of the Kemerovo region. Paleontol J. 1999;33(422–428).

[9] Panciroli E, Walsh S, Frazer NC, Brusatte SL, Corfe IJ. A reassessment of the postcanine dentition and systematics of the tritylodontid *Stereognathus* (Cynodontia, Tritylodontidae, Mammaliamorpha), from the Middle Jurassic of the UK. J Vertebr Paleontol. 2017;37(5):e1351448.

[10] Gaetano LC, Abdala F, Govender R. The postcranial skeleton of the Lower Jurassic Tritylodon longaevus from southern Africa. Ameghiniana. 2017;54:1–35.

[11] Bonaparte JF. Los tetrápodos del sector superior de la Formación Los Colorados, La Rioja, Argentina (Triásico Superior). Opera Lilloana. 1971;22:87–102.

[12] Velazco PM, Buczek AJ, Novacek MJ. Two new tritylodontids (Synapsida, Cynodontia, Mammaliamorpha) from the Upper Jurassic, southwestern Mongolia. Am Mus Novit. 2017;3874:1–35.

[13] Owen R. On the affinities of the *Stereognathus ooliticus* (Charlesworth), a mammal from the Oolitic Slate of Stonesfield. Q J Geol Soc Lond. 1857;13:1–11.

[14] Owen R. On the skull and dentition of a Triassic mammal (*Tritylodon longaevus*) from South Africa. Q J Geol Soc Lond. 1884;40:146–152.

[15] Seeley HG. On the nature and limits of reptilian characters in mammalian teeth. Proc Roy Soc. 1888;44:129–141.

[16] Simpson GG. A Catalogue of the Mesozoic Mammalia in the Geological Department of the British Museum. London1928. 215 p.

[17] Young CC. Mammal-like reptiles from Lufeng, Yunnan, China. P Zool Soc Lond. 1947;117:537–597.

[18] Kühne WG. The Liassic therapsid *Oligokyphus*. London: British Museum (Nat. Hist.); 1956.

[19] Luo Z-X, Kielan-Jaworowska Z, Cifelli RL. In quest for a phylogeny of Mesozoic mammals. Acta Palaeontol Pol. 2002;47:1–78.

[20] Crompton AW. Postcanine occlusion in cynodonts and tritylodontids. Bull Brit Mus (Nat Hist), Geol. 1972;21(2):29–71.

[21] Sues H-D. The relationships of the Tritylodontidae (Synapsida). Zool J Linn Soc. 1985;85:205–217.

[22] Hopson JA, Kitching JW. A probainognathian cynodont from South Africa and the phylogeny of nonmammalian cynodonts. Bull Mus Comp Zool. 2001;156:5–35.

[23] Bonaparte JF, Crompton AW. Origin and relationships of the Ictidosauria to non-mammalian cynodonts and mammals. Hist Biol. 2018;30:174–182.

[24] Martinez RN, May CL, Forster CA. A new carnivorous cynodont from the Ischigualasto Formation (Late Triassic, Argentina), with comments on eucynodont phylogeny. J Vertebr Paleontol. 1996;16:271–284.

[25] Ruta M, Botha-Brink J, Mitchell SA, Benton MJ. The radiation of cynodonts and the ground plan of mammalian morphological diversity. Proc R Soc B. 2013;280:20131865 10.1098/rspb.2013.1865 23986112 PMC3768321

[26] Kemp TS. Mammal-like reptiles and the origin of mammals London: Academic Press Inc.; 1982.

[27] Wible JR. Origin of Mammalia: the craniodental evidence reexamined. J Vertebr Paleontol. 1991;11:1–28.

[28] Abdala F. Redescription of *Platycraniellus elegans* (Therapsida, Cynodontia) from the Early Triassic of South Africa, and the cladistic relationships of eutheriodonts. Palaeontology. 2007;50:591–618.

[29] Liu J, Olsen P. The phylogenetic relationships of Eucynodontia. J Mammal Evol. 2010;17:151–176.

[30] Krause DW, Hoffmann S, Wible JR, Kirk EC, Schultz JA, Koenigswald Wv, et al First cranial remains of a gondwanatherian mammal reveal remarkable mosaicism. Nature. 2014;515:512–517. 10.1038/nature13922 25383528

[31] Bi S, Wang Y, Guan J, Sheng X, Meng J. Three new Jurassic euharamiyidan species reinforce early divergence of mammals. Nature. 2014;514:579 10.1038/nature13718 25209669

[32] Martinelli AG, Eltink E, Da‐Rosa AA, Langer MC. A new cynodont from the Santa Maria formation, south Brazil, improves Late Triassic probainognathian diversity. Pap Palaeontol. 2017;3:401–423.

[33] Huttenlocker AK, Grossnickle DM, Kirkland JI, Schultz JA, Luo ZX. Late-surviving stem mammal links the lowermost Cretaceous of North America and Gondwana. Nature. 2018;558:108–112. 10.1038/s41586-018-0126-y 29795343

[34] Setoguchi T, Matsuoka H, Matsuda M. New discovery of an Early Cretaceous tritylodontid (Reptilia, Therapsida) from Japan and the phylogenetic reconstruction of Tritylodontidae based on the dental characters. In: Wang Y, Deng T, editors. Proceedings of the Seventh Annual Meeting of the Chinese Society of Vertebrate Paleontology1999. p. 117–124.

[35] Lazzari V, Schultz JA, Tafforeau P, Martin T. Occlusal pattern in paulchoffatiid multituberculates and the evolution of cusp morphology in mammaliamorphs with rodent-like dentitions. J Mammal Evol. 2010;17:177–192.

[36] Jasinoski SC, Chinsamy A. Mandibular histology and growth of the nonmammaliaform cynodont *Tritylodon*. J Anat. 2012;220:564–579.

[37] Sues H-D. The skull and dentition of two tritylodontid synapsids from the Lower Jurassic of western North America. Bull Mus Comp Zool. 1986;151:217–268.

[38] Selby PJ, Babington CC, Balfour JH, Taylor R. On the Stereognathus Ooliticus, from the Stonesfield Slate. [Comment on Owen 1857]. Ann Mag Nat Hist. 1857;19:103–104.

[39] Hu Y, Meng J, Clark JM. A new tritylodontid from the Upper Jurassic of Xinjiang, China. Acta Palaeontol Pol. 2009;54:385–391.

[40] Kemp TS. The Origin and Evolution of Mammals. Oxford: Oxford University Press 2005.

[41] Sues H-D. Inferences concerning feeding and locomotion in the Tritylodontidae (Synapsida). In: Reiff WE, Westphal F, editors. Third Symposium on Terrestrial Ecosystems, short papers1984. p. 231–236.

[42] O’Meara RN, Asher RJ. The evolution of growth patterns in mammalian versus nonmammalian cynodonts. Paleobiology. 2016;42:439–464.

[43] Kalthoff DC, Green JL. Feeding ecology in Oligocene mylodontoid sloths (Mammalia, Xenarthra) as revealed by orthodentine microwear analysis. J Mammal Evol. 2018;25:551–564. 10.1007/s10914-017-9405-x 30443148 PMC6209052

[44] King T, Andrews P, Boz B. Effect of taphonomic processes on dental microwear. Am J Phys Anthropol. 1999;108:359–373. 10.1002/(SICI)1096-8644(199903)108:3<359::AID-AJPA10>3.0.CO;2-9 10096686

[45] Solounias N, Semprebon GM. Advances in the reconstruction of ungulate ecomorphology with application to early fossil equids. Am Mus Novit. 2002;3366:1–49.

[46] Schulz E, Calandra I, Kaiser TM. Applying tribology to teeth of hoofed mammals. Scanning. 2010;32:162–182. 10.1002/sca.20181 20949615

[47] Hammer Ø, Harper DAT, Ryan PD. PAST: Paleontological Statistics Software Package for Education and Data Analysis. Palaeontol Electron. 2001;4:1–9.

[48] Godfrey LR, Semprebon GM, Jungers WL, Sutherland MR, Simons EL, Solounias N. Dental use wear in extinct lemurs: evidence of diet and niche differentiation. J Hum Evol. 2004;47:145–169. 10.1016/j.jhevol.2004.06.003 15337413

[49] Schulz E, Piotrowski V, Clauss M, Mau M, Merceron G, Kaiser TM. Dietary abrasiveness is associated with variability of microwear and dental surface texture in rabbits. PLoS ONE. 2013;8:e56167 10.1371/journal.pone.0056167 23405263 PMC3566079

[50] Calandra I, Labonne G, Schulz-Kornas E, Kaiser TM, Montuire S. Tooth wear to quantify intra-specific variations in diet and chewing mechanics. Sci Rep. 2016;6:34037 10.1038/srep34037 27658531 PMC5034321

[51] Kaiser TM, Clauss M, Schulz-Kornas E. A set of hypotheses on tribology of mammalian herbivore teeth. Surf Topogr: Metrol Prop. 2016;4:014003.

[52] Mihlbachler MC, Rivals F, Solounias N, Semprebon GM. Dietary change and evolution of horses in North America. Science. 2011;331:1178–1181. 10.1126/science.1196166 21385712

[53] Schulz E, Calandra I, Kaiser TM. Feeding ecology and chewing mechanics in hoofed mammals: 3D tribology of enamel wear. Wear. 2013;300:169–179.

[54] Gailer JP, Calandra I, Schulz-Kornas E, Kaiser TM. Morphology is not destiny: discrepancy between form, function and dietary adaptation in bovid check teeth. J Mammal Evol. 2016;23:369–383.

[55] Gomes Rodrigues H, Merceron G, Viriot L. Dental microwear patterns of extant and extinct Muridae (Rodentia, Mammalia): ecological implications. Naturwissenschaften. 2009;96:537–542. 10.1007/s00114-008-0501-x 19127354

[56] Winkler DE, Andrianasolo T, Andriamandimbiarisoa L, Ganzhorn J, Rakotondranary J, Kaiser TM, et al Tooth wear patterns in black rats (*Rattus rattus*) of Madagascar differ more in relation to human impact than to differences in natural habitats. Ecol Evol. 2016;6:2205–2215. 10.1002/ece3.2048 27069577 PMC4782253

[57] Goswami A, Flynn JJ, Ranivoharimanana L, Wyss AR. Dental microwear in Triassic amniotes: Implications for paleoecology and mastication mechanics. J Vertebr Paleontol. 2005;25:320–329.

[58] Kubo T, Yamada E, Kubo MO. Masticatory jaw movement of Exaeretodon argentinus (Therapsida: Cynodontia) inferred from its dental microwear. PLoS ONE 2017;12(11):e0188023 10.1371/journal.pone.0188023 29186178 PMC5706674

[59] Strait SG. Differences in occlusal morphology and molar size in frugivores and faunivores. J Hum Evol. 1993;25:471–484.

[60] Evans AR, Sanson GD. The effect of tooth shape on the breakdown of insects. J Zool. 1998;246:391–400.

[61] Evans AR, Sanson GD. Biomechanical properties of insects in relation to insectivory: cuticule thickness as an indicator of insect ‘hardness’ and ‘intractability’. Aust J Zool. 2005;53:9–19.

[62] Gliwicz J. Niche segregation in a rodent community of African dry savanna. J Mamm. 1987;68:169–172.

[63] Gill PG, Purnell MA, Crumpton N, Robson Brown K, Gostling NJ, Stampanoni M, et al Dietary specializations and diversity in feeding ecology of the earliest stem mammals. Nature. 2014;512:303–305. 10.1038/nature13622 25143112

[64] Purnell MA, Crumpton N, Gill PG, Rayfield EJ. Within-guild dietary discrimination from 3-D textural analysis of tooth microwear in insectivorous mammals. J Zool. 2013;291:249–257. 10.1111/jzo.12068 PMC4296236 25620853

[65] Friis EM, Crane PR, Pederson KR. The early flowers and angiosperm evolution Cambridge: Cambridge University Press; 2011.

[66] McLoughlin S, Pott C. Plant mobility in the Mesozoic: disseminule dispersal strategies of Chinese and Australian Middle Jurassic to Early Cretaceous plants. Palaeogeogr Palaeoclimatol Palaeoecol. 2018 10.1016/j.palaeo.2018.10.003

[67] Grimaldi D, Engel MS. Evolution of the insects New York: Cambridge University Press; 2005.

[68] Hoffmann EA, Rowe TB. Postcranial anatomy of *Kayentatherium wellesi*: swimming adaptations in a mammaliamorph from the Early Jurassic. J Vertebr Paleontol Progr Abstr. 2017;2017:130.

[69] Gray J. Evolution of the freshwater ecosystem: The fossil record. Palaeogeogr Palaeoclimatol Palaeoecol. 1988;62:1–214.

[70] Martín-Closas C. The fossil record and evolution of freshwater plants: a review. Geol Acta. 2003;1:315–338.

