# Supplementary figures and images for: Complementary approaches to tooth wear analysis in Tritylodontidae (Synapsida, Mammaliamorpha) reveal a generalist diet
Source: PLoS One. 2019 Jul 25;14(7):e0220188. doi: 10.1371/journal.pone.0220188 (PMC6658083; doi:10.1371/journal.pone.0220188)

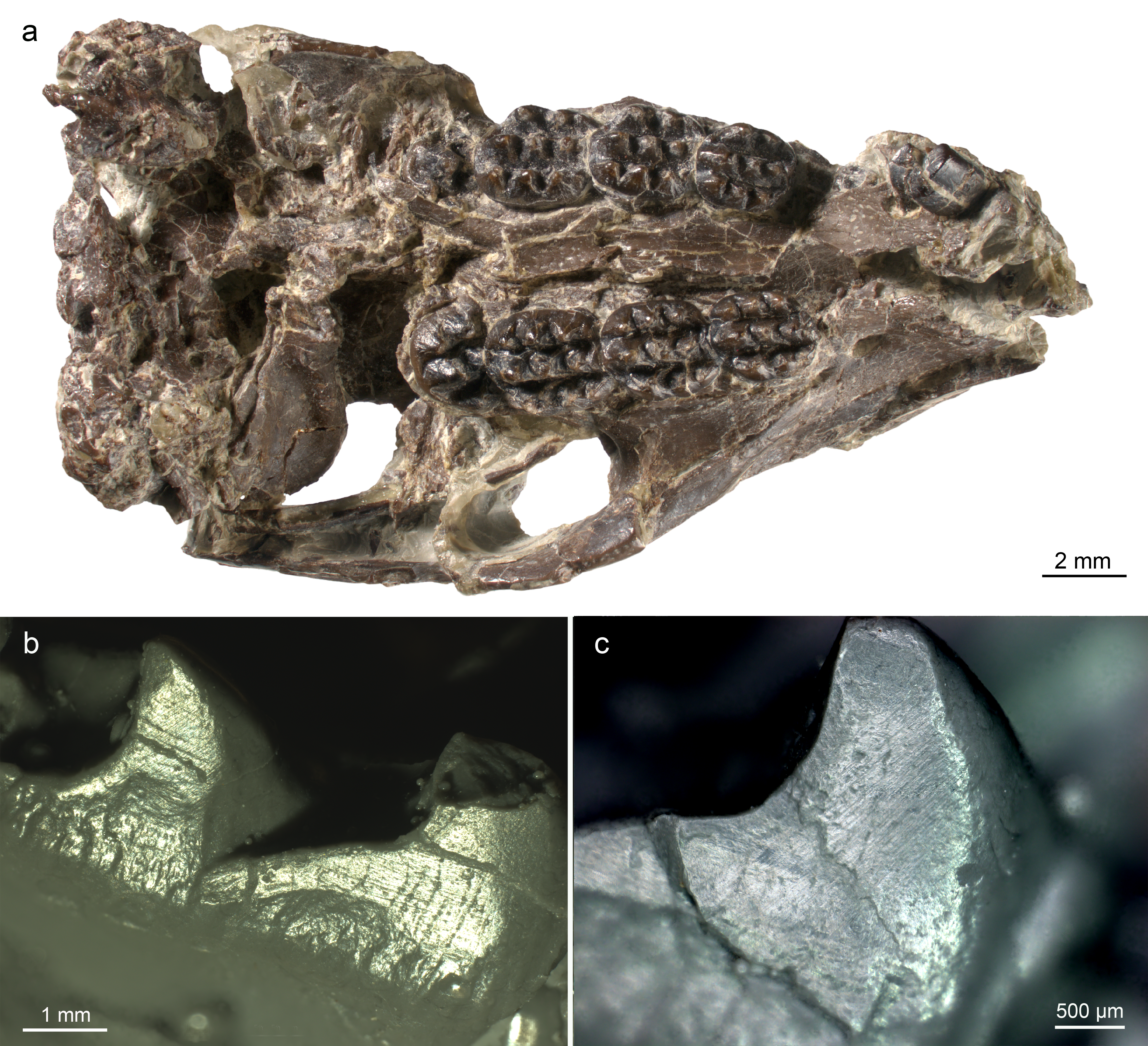

Supplement: S1 Fig — (a) Skull of Oligokyphus sp. (MCZ 8843) in ventral view. This specimen was excluded from analysis because of postmortem alterations on the wear facets. (b) and (c) Wear facets (on high-resolution casts) of lower postcanines of Kayentatherium wellesi (MCZ 8811). In all three images, anterior is to the right. (TIF) [file pone.0220188.s001.tif]
