# Supplementary material for: Complementary approaches to tooth wear analysis in Tritylodontidae (Synapsida, Mammaliamorpha) reveal a generalist diet
Source: PLoS One. 2019 Jul 25;14(7):e0220188. doi: 10.1371/journal.pone.0220188 (PMC6658083; doi:10.1371/journal.pone.0220188)
Supplement: S1 Table — Country codes refer to ISO 3166–1 alpha-2. Abbreviations: L, large-bodied; M, medium-bodied; pc, lower postcanine; PC, upper postcanine; S, small-bodied. Acronyms to museum collections: BRSUG: Geology Museum, University of Bristol, Bristol, GB; FMNH: Finnish Museum of Natural History, Helsinki, Finland; GLRCM: Gloucester City Museum, Gloucester, GB; GPIT: Paläontologische Sammlung, Fachbereich Geowissenschaften, Universität Tübingen, DE; MCZ: Museum of Comparative Zoology, Harvard, Massachusetts, USA; NHMUK: Natural History Museum, London, GB; USNM PAL: National Museum of Natural History, Department of Paleobiology, Washington D.C., USA; ZIN PH: Paleoherpetological collection of the Zoological Institute, Russian Academy of Sciences, Saint Petersburg, RU. (PDF) [file pone.0220188.s002.pdf]

S1 Table. Specimen details of the tritylodontid taxa analysed.

Taxon (body size estimation)	Collection ID	Tooth position	Age	Locality (as on specimen label)
Oligokyphus major (S)	BRSUG 26300	pc 1-3	Early Jurassic (Pliensbachian)	Windsor Hill, GB
Oligokyphus major (S)	BRSUG 26295_D	pc (anterior fragment)	Early Jurassic (Pliensbachian)	Windsor Hill, GB
Oligokyphus sp. (S)	NHMUK PV R 37376	?pc	Early Jurassic (Hettangian)	Pant 4 quarry, St. Brides Island, GB
Oligokyphus sp. (S)	MCZ 111 AR 83	pc	Early Jurassic (Pliensbachian)	Kayenta Fm., Adeli Echii Cliffs, Gold Spring Quarry, Coconino County, Arizona, US
Oligokyphus triserialis (S)	GPIT-1577-8	PC	Upper Triassic (Rhaetian)	Olgahain, DE
Tritylodon longaevus (L)	FMNH P30036	PC5 dext.	Early Jurassic	South Africa
Stereognathus ooliticus (M)	GLRCM H38-9	?pc	Middle Jurassic (Early Bathonian)	Hornsleasow, GB
Stereognathus ooliticus (M)	GLRCM 75710	PC ?sin.	Middle Jurassic (Early Bathonian)	Hornsleasow, GB
Stereognathus ooliticus (M)	GLRCM 2104	PC dext.	Middle Jurassic (Early Bathonian)	Hornsleasow, GB
Stereognathus ooliticus (M)	GLRCM H174	pc	Middle Jurassic (Early Bathonian)	Hornsleasow, GB
Stereognathus ooliticus (M)	GLRCM TRAY-12-22	pc	Middle Jurassic (Early Bathonian)	Hornsleasow, GB
Stereognathus cf. *ooliticus* (M)	FMNH P30037	pc (fragment)	Middle Jurassic (Late Bathonian)	Old Cement Works Quarry, Kirtlington, Oxfordshire, UK
Kayentatherium wellesi (L)	MCZ 8837	pc sin.	Early Jurassic (Pliensbachian)	Kayenta Fm. , Adeli Echii Cliffs, Gold Spring Quarry, Coconino County, Arizona, US
Kayentatherium wellesi (L)	MCZ 8811	PC2 sin., PC3 dext., pc3 dext.	Early Jurassic (Pliensbachian)	Kayenta Fm. (M. Silty facies), Little Colorado River Valley - Ward Terr.; Rock Head Loc., Coconino County, Arizona, US
Kayentatherium wellesi (L)	USNM PAL 317208	pc1	Early Jurassic (Pliensbachian)	Kayenta Fm., US
Dinnebitodon amarali (M)	MCZ 8830-1	PC2 sin.	Early Jurassic (Pliensbachian)	Kayenta Fm. (silty facies), Little Colorado River Valley - Adeii Echii Cliffs; Gold Springs Wash, Coconino County, Arizona, US

Dinnebitodon amarali (M)	MCZ 8831	PC2 sin., PC2 dext.	Early Jurassic (Pliensbachian)	Kayenta Fm. (silty facies), Adeii Echii Cliffs; Gold Spring off cow trail, Coconino County, Arizona, US
cf. *Dinnebitodon amarali* (M)	MCZ 4 (8931)	PC2	Early Jurassic (Pliensbachian)	Kayenta Fm. (silty facies), Adeli Echii Cliffs, Gold Spring Quarry, Coconino County, Arizona, US
Stereognathus sp. (M)	ZIN PH 63/117	PC/pc (fragment)	Middle Jurassic (Bathonian)	Itat Fm. (I2), Berezovsk Quarry, Krasnoyarsk, RU
Stereognathus sp. (M)	ZIN PH 64/117	PC/pc (fragment)	Middle Jurassic (Bathonian)	Itat Fm. (I2), Berezovsk Quarry, Krasnoyarsk, RU
Stereognathus sp. (M)	ZIN PH 65/117	?PC (fragment)	Middle Jurassic (Bathonian)	Itat Fm. (I2), Berezovsk Quarry, Krasnoyarsk, RU
Stereognathus sp. (M)	ZIN PH 66/117	pc	Middle Jurassic (Bathonian)	Itat Fm. (I2), Berezovsk Quarry, Krasnoyarsk, RU
Stereognathus sp. (M)	ZIN PH 67/117	pc (posterior fragment)	Middle Jurassic (Bathonian)	Itat Fm. (I2), Berezovsk Quarry, Krasnoyarsk, RU
Stereognathus sp. (M)	ZIN PH 68/117	pc (posterior fragment)	Middle Jurassic (Bathonian)	Itat Fm. (I2), Berezovsk Quarry, Krasnoyarsk, RU
Stereognathus sp. (M)	ZIN PH 69/117	PC (fragment)	Middle Jurassic (Bathonian)	Itat Fm. (I2), Berezovsk Quarry, Krasnoyarsk, RU
Stereognathus sp. (M)	ZIN PH 70/117	PC/pc	Middle Jurassic (Bathonian)	Itat Fm. (I2), Berezovsk Quarry, Krasnoyarsk, RU
Stereognathus sp. (M)	ZIN PH 71/117	PC/pc	Middle Jurassic (Bathonian)	Itat Fm. (I2), Berezovsk Quarry, Krasnoyarsk, RU
Stereognathus sp. (M)	ZIN PH 72/117	pc (anterior fragment)	Middle Jurassic (Bathonian)	Itat Fm. (I2), Berezovsk Quarry, Krasnoyarsk, RU
Stereognathus sp. (M)	ZIN PH 73/117	PC/pc	Middle Jurassic (Bathonian)	Itat Fm. (I2), Berezovsk Quarry, Krasnoyarsk, RU
Stereognathus sp. (M)	ZIN PH 74/117	PC/pc	Middle Jurassic (Bathonian)	Itat Fm. (I2), Berezovsk Quarry, Krasnoyarsk, RU
Stereognathus sp. (M)	ZIN PH 75/117	PC	Middle Jurassic (Bathonian)	Itat Fm. (I2), Berezovsk Quarry, Krasnoyarsk, RU
Stereognathus sp. (M)	ZIN PH 76/117	PC/pc (fragment)	Middle Jurassic (Bathonian)	Itat Fm. (I2), Berezovsk Quarry, Krasnoyarsk, RU
Stereognathus sibiricus (M)	ZIN PH 3/154	PC/pc (fragment)	Early Cretaceous (Barremian-Aptian)	Ilek Fm., W1-Shestakovo 1, RU
Stereognathus sibiricus (M)	ZIN PH 4/154	pc	Early Cretaceous (Barremian-Aptian)	Ilek Fm., W1-Shestakovo 1, RU
Stereognathus sibiricus (M)	ZIN PH 5/154	PC	Early Cretaceous (Barremian-Aptian)	Ilek Fm., W1-Shestakovo 1, RU
Stereognathus sibiricus (M)	ZIN PH 6/154	pc	Early Cretaceous (Barremian-Aptian)	Ilek Fm., W1-Shestakovo 1, RU
Stereognathus sibiricus (M)	ZIN PH 7/154	pc	Early Cretaceous (Barremian-Aptian)	Ilek Fm., W1-Shestakovo 1, RU

Stereognathus sibiricus (M)	ZIN PH 8/154	?pc	Early Cretaceous (Barremian-Aptian)	Ilek Fm., W1-Shestakovo 1, RU
Stereognathus sibiricus (M)	ZIN PH 9/154	PC	Early Cretaceous (Barremian-Aptian)	Ilek Fm., W1-Shestakovo 1, RU

Country codes refer to ISO 3166-1 alpha-2. Abbreviations: L, large-bodied; M, medium-bodied; pc, lower postcanine; PC, upper postcanine; S, small-bodied. Acronyms to museum collections: BRSUG: Geology Museum, University of Bristol, Bristol, GB; FMNH: Finnish Museum of Natural History, Helsinki, Finland; GLRCM: Gloucester City Museum, Gloucester, GB; GPIT: Paläontologische Sammlung, Fachbereich Geowissenschaften, Universität Tübingen, DE; MCZ: Museum of Comparative Zoology, Harvard, Massachusetts, USA; NHMUK: Natural History Museum, London, GB; USNM PAL: National Museum of Natural History, Department of Paleobiology, Washington D.C., USA; ZIN PH: Paleoherpetological collection of the Zoological Institute, Russian Academy of Sciences, Saint Petersburg, RU.
