# Supplementary material for: Complementary approaches to tooth wear analysis in Tritylodontidae (Synapsida, Mammaliamorpha) reveal a generalist diet
Source: PLoS One. 2019 Jul 25;14(7):e0220188. doi: 10.1371/journal.pone.0220188 (PMC6658083; doi:10.1371/journal.pone.0220188)
Supplement: S2 Table — Numerical parameters of stereoscopic microwear (small pits, large pits, fine scratches, and coarse scratches) and parameters of 3D surface texture (pit void volume, peak density, peak curvature, and dale area) are shown. Significant p-values are in bold. (PDF) [file pone.0220188.s003.pdf]

S2 Table. ANOVA test results of tritylodontid species with N ≥ 2.

		Sum of sqrs	df	Mean sqrs	F	p
	Between groups:	829 686	5	165 937	43.44	**1.34E-09**
Small pits	Within groups:	110 781	29	382 003		
	Total:	940 466	34			
	Between groups:	914 546	5	182 909	6 342	**0.0004308**
Large pits	Within groups:	836 379	29	288 406		
	Total:	175 092	34			
	Between groups:	346 522	5	693 045	15.29	**2.23E-04**
Fine scratches	Within groups:	131 405	29	453 119		
	Total:	477 927	34			
	Between groups:	566 271	5	113 254	5 436	**0.0012**
Coarse scratches	Within groups:	604 236	29	208 357		
	Total:	117 051	34			
	Between groups:	0.135	5	0.027	0.91	0.4858
Pit void volume	Within groups:	0.859	29	0.03		
	Total:	0.995	34			
	Between groups:	1321	5	264.2	2.76	**0.0368**
Peak density	Within groups:	2772	29	95.59		
	Total:	4093	34			
	Between groups:	1045	5	208.9	1.92	0.1216
Peak curvature	Within groups:	3157	29	108.9		
	Total:	4202	34			
	Between groups:	1.008	5	0.202	1.12	0.3699
Dale area	Within groups:	5.203	29	0.179		
	Total:	6.211	34			

Numerical parameters of stereoscopic microwear (small pits, large pits, fine scratches, and coarse scratches) and parameters of 3D surface texture (pit void volume, peak density, peak curvature, and dale area) are shown. Significant p-values are in bold.
