# Supplementary material for: Complementary approaches to tooth wear analysis in Tritylodontidae (Synapsida, Mammaliamorpha) reveal a generalist diet
Source: PLoS One. 2019 Jul 25;14(7):e0220188. doi: 10.1371/journal.pone.0220188 (PMC6658083; doi:10.1371/journal.pone.0220188)
Supplement: S3 Table — Abbreviations (as ordered in table): SP = small pits; LP = large pits; FS = fine scratches; CS = coarse scratches; Spd_rank = peak density (rank-transformed); Spc_rank = arithmetic mean peak curvature (rank-transformed); Sda_log = mean dale area (log-transformed); Vvv_log = pit void volume (log-transformed). (PDF) [file pone.0220188.s004.pdf]

**S3 Table. PCA variances and loadings of the significant parameter variables for stereoscopic microwear and 3D surface texture**.

| **Stereoscopic microwear** | | | | |
|---|---|---|---|---|
| PC | Eigenvalue | % variance | | |
| 1 | 35.8009 | 74.546 | | |
| 2 | 7.15454 | 14.897 | | |
| 3 | 3.59715 | 7.4901 | | |
| 4 | 1.47264 | 3.0664 | | |
| | PC 1 | PC 2 | PC 3 | PC 4 |
| SP | 0.82265 | -0.47986 | 0.27989 | 0.12097 |
| LP | 0.1935 | 0.58546 | 0.63571 | -0.4644 |
| FS | 0.52447 | 0.44157 | -0.70338 | -0.18763 |
| CS | 0.10356 | 0.48165 | 0.15098 | 0.85703 |
| **3D texture analysis** | | | | |
| PC | Eigenvalue | % variance | | |
| 1 | 158.711 | 61.008 | | |
| 2 | 101.232 | 38.913 | | |
| 3 | 0.182443 | 0.070131 | | |
| 4 | 0.0219985 | 0.0084562 | | |
| | PC 1 | PC 2 | PC 3 | PC 4 |
| *Spd*_rank | 0.70646 | -0.70768 | 0.01041 | -0.0013664 |
| *Spc*_rank | 0.7076 | 0.70652 | 0.008353 | -0.0070941 |
| *Sda*_log | -0.012092 | 0.0021149 | 0.98611 | 0.16561 |
| *Vvv*_log | 0.0080998 | 0.0037468 | -0.16553 | 0.98616 |

Abbreviations (as ordered in table): SP = small pits; LP = large pits; FS = fine scratches; CS = coarse scratches; *Spd*_rank = peak density (rank-transformed); *Spc*_rank = arithmetic mean peak curvature (rank-transformed); *Sda*_log = mean dale area (log-transformed); *Vvv*_log = pit void volume (log-transformed)
