# Supplementary material for: Complementary approaches to tooth wear analysis in Tritylodontidae (Synapsida, Mammaliamorpha) reveal a generalist diet
Source: PLoS One. 2019 Jul 25;14(7):e0220188. doi: 10.1371/journal.pone.0220188 (PMC6658083; doi:10.1371/journal.pone.0220188)
Supplement: S4 Table — (PDF) [file pone.0220188.s005.pdf]

S4 Table. Key characters of major, widely distributed, Mesozoic plant groups

Plant group	Stature	Specialized starch storage structures	Main reproductive organs	Primary seed dispersal strategy	Stratigraphic range
Pinales (conifers)	Woody shrubs to tall trees	None	Leathery to woody cones, less commonly fleshy-coated or fleshy-supported structures (arils, epimatia, expanded receptacles); seeds small (<1 mm) to large (>50 mm)	Variable: many adopt anemochory, some utilize endochory	Carboniferous to present
Ginkgoales	Woody trees	None	Medium to large seeds with a hard inner coat and a fleshy outer coat	Barochory or endochory	Permian to present
Leptostrobales (Czekanowskiales)	Woody trees?	None	Capsule like organs enclosing small unspecialized seeds	Barochory?	Late Triassic to Cretaceous
Umkomasiales (corystosperms)	Woody trees	None	Multi-ovulate branched structures with medium-sized seeds enclosed in leathery or fleshy cupules	Barochory, anemochory or endochory?	Latest Permian to Cretaceous (relict species persisting to Eocene)
Peltaspermales	Woody shrubs or trees	None	Multi-ovulate branched structures with medium-sized seeds borne on leathery peltate cupules	Barochory	Permian to Triassic (relict species persisting to end of Jurassic)
Caytoniales	Woody shrubs or trees?	None	Multi-ovulate branched structures with small seeds enclosed in leathery or fleshy cupules	Barochory or endochory?	Triassic to Cretaceous

Bennettitales	Divaricate-branched woody shrubs and stocky monopodial shrubs	Potentially specialized storage in pith or cortex of cycadeoid bennettites	Flower-like structures bearing many small seeds	Anemochory?	Latest Permian to Cretaceous (relict species persisting to Oligocene)
Nilssoniales	Thin-branched woody shrubs	None	Medium-sized, hard-coated seeds	Barochory or endochory?	Triassic to Cretaceous
Pentoxylales	Thin-branched woody shrubs to small trees	None	Small, hard-coated seeds	Barochory or endochory?	Triassic to Cretaceous (restricted to Gondwana?)
Cycadales	Stocky monopodial shrubs	Large pith in manoxylic stem	Large seeds with leathery to hard coatings	Barochory or endochory?	Carboniferous to present
Gnetales	Climbing woody shrubs, small wiry shrubs and Stocky monopodial shrubs	Large root system in some taxa	Small to medium, hard, papery or fleshy seeds borne in cones, or large nut-like seeds	Variable: Endochory, anemochory, barochory	Triassic to present
Ferns	Rhizomatous herbs, epiphytes and monopodial shrubs	Enlarged rhizomes and bulbils in some species	Leaf-borne sporangia	Wind-dispersed spores	Devonian to present
Equisetales	Reed-like Rhizomatous herbs to monopodial shrubs with	Enlarged rhizomes and bulbils	Sporangia borne in terminal strobili	Wind- and water-dispersed spores	Devonian to present

	silica-rich stems	in some species			
Lycopsida	Rhizomatous herbs, epiphytes and monopodial herbs to shrubs	Enlarged corm-lik rhizophore in some groups	Sporangia borne in terminal strobili	Wind- and water-dispersed spores	Devonian to present
